# Pharmacoproteomics of Brain Barrier Transporters and Substrate Design for the Brain Targeted Drug Delivery

**DOI:** 10.1007/s11095-022-03193-2

**Published:** 2022-03-07

**Authors:** Kristiina M. Huttunen, Tetsuya Terasaki, Arto Urtti, Ahmed B. Montaser, Yasuo Uchida

**Affiliations:** 1 School of Pharmacy, Faculty of Health Sciences, University of Eastern Finland, P.O. Box 1627, FI-70211 Kuopio, Finland; 2 Graduate School of Pharmaceutical Sciences, Tohoku University, 6-3 Aoba, Aramaki, Aoba-ku, Sendai, 980-8578 Japan

**Keywords:** blood-arachnoid barrier, blood-brain barrier, molecular dynamics simulations, pharmacoproteomics, prodrug

## Abstract

One of the major reasons why central nervous system (CNS)-drug development has been challenging in the past, is the barriers that prevent substances entering from the blood circulation into the brain. These barriers include the blood-brain barrier (BBB), blood-spinal cord barrier (BSCB), blood-cerebrospinal fluid barrier (BCSFB), and blood-arachnoid barrier (BAB), and they differ from each other in their transporter protein expression and function as well as among the species. The quantitative expression profiles of the transporters in the CNS-barriers have been recently revealed, and in this review, it is described how they affect the pharmacokinetics of compounds and how these expression differences can be taken into account in the prediction of brain drug disposition in humans, an approach called *pharmacoproteomics*. In recent years, also structural biology and computational resources have progressed remarkably, enabling a detailed understanding of the dynamic processes of transporters. Molecular dynamics simulations (MDS) are currently used commonly to reveal the conformational changes of the transporters and to find the interactions between the substrates and the protein during the binding, translocation in the transporter cavity, and release of the substrate on the other side of the membrane. The computational advancements have also aided in the rational design of transporter-utilizing compounds, including prodrugs that can be actively transported without losing potency towards the pharmacological target. In this review, the state-of-art of these approaches will be also discussed to give insights into the transporter-mediated drug delivery to the CNS.

**Supplementary Information:**

The online version contains supplementary material available at 10.1007/s11095-022-03193-2.

## Introduction

The discovery and development of new drugs acting on the central nervous system (CNS) is a crucial subject in today’s aging society. It is well known that the success rate of CNS-acting drug development is significantly low (1–3). One of the major reasons is that the blood-brain barrier (BBB) restricts the drug entry from the circulating blood to the brain. In recent years, several transport carrier proteins expressed in the BBB have been quantified by mass spectrometry and the differences in animal species have been clarified (4–9).

Recently, the usefulness and importance of modeling and simulation have also been well recognized for drug development (10–12). Although the processes of drug absorption, distribution, metabolism, and excretion are greatly governed by the function of carrier-mediated transporters and metabolic enzymes, the expression levels of the responsible proteins are not fully utilized in modeling and simulation. Quantification of transporters in the brain barriers facilitates modeling and simulation towards *in vitro - vivo* extrapolation (IVIVE), thereby enabling the rational design of drugs that are capable of penetrating the BBB (13–15).

However, designing and developing compounds that can take advantage of transporter-mediated delivery across the BBB has been challenging, mainly because until very recently protein structures available for molecular modelers have not been with high enough resolution. Moreover, another challenge is to combine the structural features required for the potency together with features required for the interactions with the target transporter. Hence, a prodrug approach has been utilized in many cases to create transporter substrate mimicking bioreversible conjugates.

In recent years, the progress in structural biology, computational modeling, and data science have been remarkable, and many new findings have been reported in the structural analysis of cell membrane transporter proteins (16–18). Since the transport carriers dynamically change their conformations to exert their function, the relationship between the substrate and the transport activity based on the crystal structure can be limited. On the other hand, Molecular Dynamics Simulations (MDS) can enable clarification of the dynamic structural changes of transporter proteins and elucidate the substrate recognition and translocation through the protein cavity (19–21).

In this review, we will summarize; 1) the similarity and differences of four CNS-barriers, 2) determinant factors affecting the drug concentration in the cerebrospinal fluid (CSF), and 3) usefulness of units of pmol/g of whole tissue weight containing barrier cells and fmol/cm^2^ of surface area containing barrier cell layer. Further, 4) the advantages and significance of prodrug design for the CNS-acting drugs will be discussed together with 5) the importance of drug structural design by MDS for the substrate of the target transporter.

## Barriers of the Central Nervous System (CNS)

### Four Barriers Play Crucial Roles in Substrate Exchange between the Circulating Blood and the Separated CNS Region Independently

**Fig. 1:**
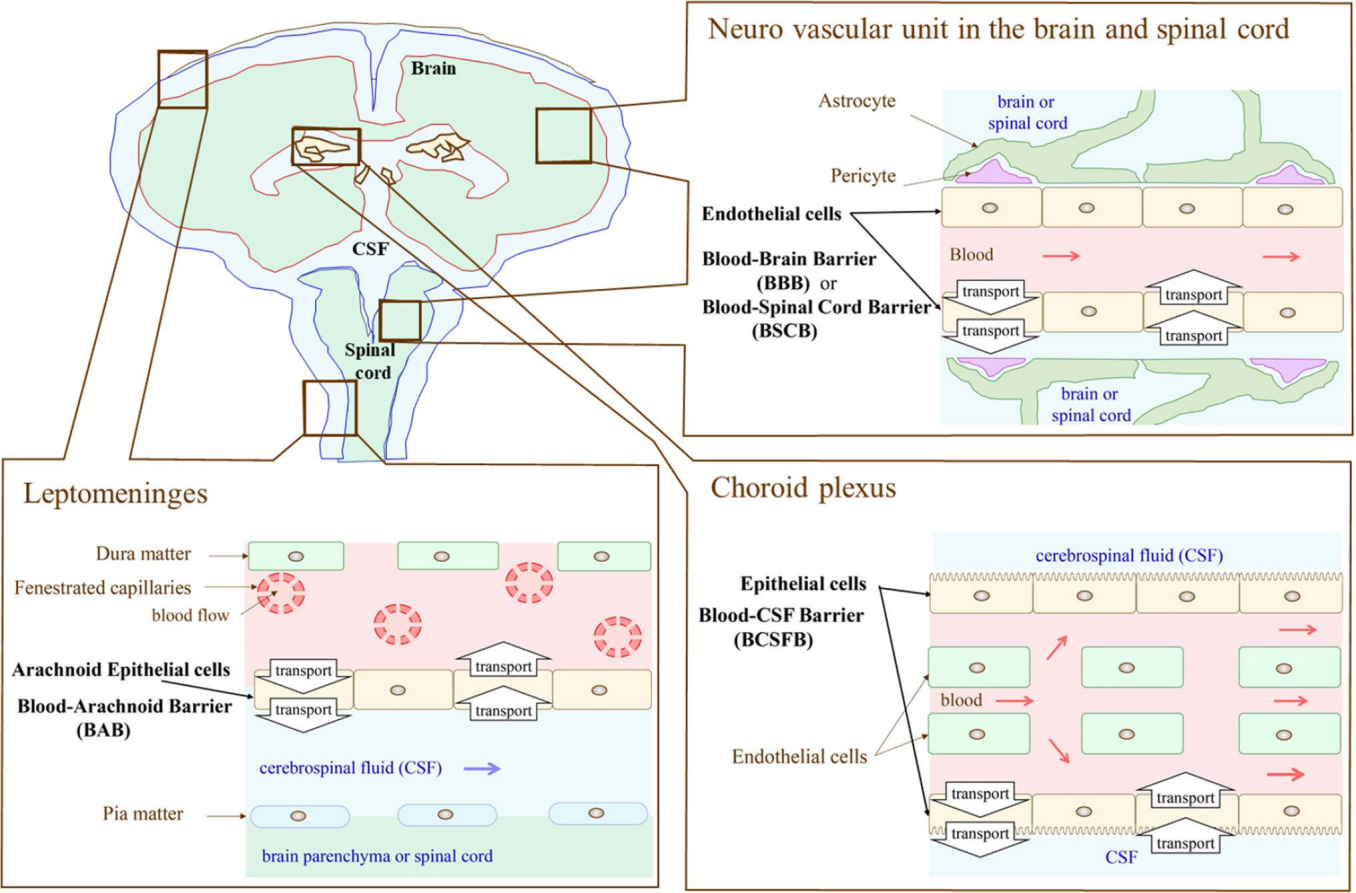
Anatomical structure of barriers of the Central Nervous System.

As illustrated in Fig. 1, there are four barriers in the CNS. The blood-brain barrier (BBB), consisting of brain capillary endothelial cells, plays a major role in substrate exchange between circulating blood (flow rate in humans: 700 mL/min) and the extracellular fluid of the brain parenchyma (weight in human: 1400 g) (22). The volume of brain capillary endothelial cells consists of 0.1% in the brain, while the surface area is 12 m^2^. Small molecules will reach glia and neurons rapidly by passive diffusion after crossing the BBB, as the average distance between neighboring capillaries is 45 μm, which is sufficiently short. Nevertheless, the diffusion rate in the brain parenchyma is significantly restricted for longer distances, e.g., 1 mm or longer. The blood-spinal cord barrier (BSCB), consisting of capillary endothelial cells in the spinal cord, plays a major role in substrate exchange between the circulating blood and the extracellular fluid of spinal cord tissue. The blood-cerebrospinal fluid barrier (BCSFB), consisting of choroid plexus epithelial cells, plays a major role in cerebrospinal fluid (CSF) production (flow rate in humans: about 350 μL/min) and substrate exchange between the circulating blood and the CSF. CSF located in the ventricular space has a volume of 25 mL in humans, i.e., 18% of the total amount of CSF (23). The blood-arachnoid barrier (BAB), consisting of arachnoid epithelial cells, plays a major role in substrate exchange between the circulating blood and the CSF in the subarachnoid space (volume in human is 115 mL, i.e., 82% of total CSF amount) (24). All cell layers consisting of barriers are connected with tight junctions, thereby substrate exchange is limited to the transcellular pathway in the CNS barriers. Notably, the ependymal cell layer responsible for the exchange of brain interstitial fluids (ISF) and circulating CSF, consists in turn, of leaky gap junctions allowing compounds with large molecular weight to permeate through the monolayer located at the apical surface of ventricular space (25). Therefore, characterization of the transport system in the respective barriers and comparison of the plasma membrane permeability rate between carrier-mediated transport and passive diffusion are important subjects for the drug delivery to the CNS.

### Drug Concentration in the CSF Is Not a Surrogate of the BBB Permeability

Considering drug effects in the CNS, unbound drug concentration in the extracellular fluid of the CNS is the most relevant concentration. K_p,uu,brain_ defined by the AUC (area under the drug concentration) of unbound drug concentration ratio between brain and blood (Eq. 1) from time zero to infinity is governed by the ratio of permeability rate across the BBB between influx rate (from blood to the brain) and efflux rate (from the brain to blood) (26, 27). Therefore, K_p,uu,brain_ is one of the most valuable parameters evaluating the differential BBB permeability for different drugs, animal species, and diseased conditions.

1 $${\mathrm{K}}_{\mathrm{p},\mathrm{uu},\mathrm{brain}}=\frac{{\mathrm{AUC}}_{\mathrm{u},\mathrm{brain},0-\mathrm{infinity}}}{{\mathrm{AUC}}_{\mathrm{u},\mathrm{blood},0-\mathrm{infinity}}}$$

2 $$=\frac{\mathrm{Influx}\ \mathrm{permeability}\ \mathrm{rate}\ \mathrm{from}\ \mathrm{blood}\ \mathrm{to}\ \mathrm{the}\ \mathrm{brain}\ \mathrm{across}\ \mathrm{the}\ \mathrm{BBB}\ }{\mathrm{Efflux}\ \mathrm{permeability}\ \mathrm{rate}\ \mathrm{from}\ \mathrm{the}\ \mathrm{brain}\ \mathrm{to}\ \mathrm{blood}\ \mathrm{across}\ \mathrm{the}\ \mathrm{BBB}}$$

**Fig. 2:**
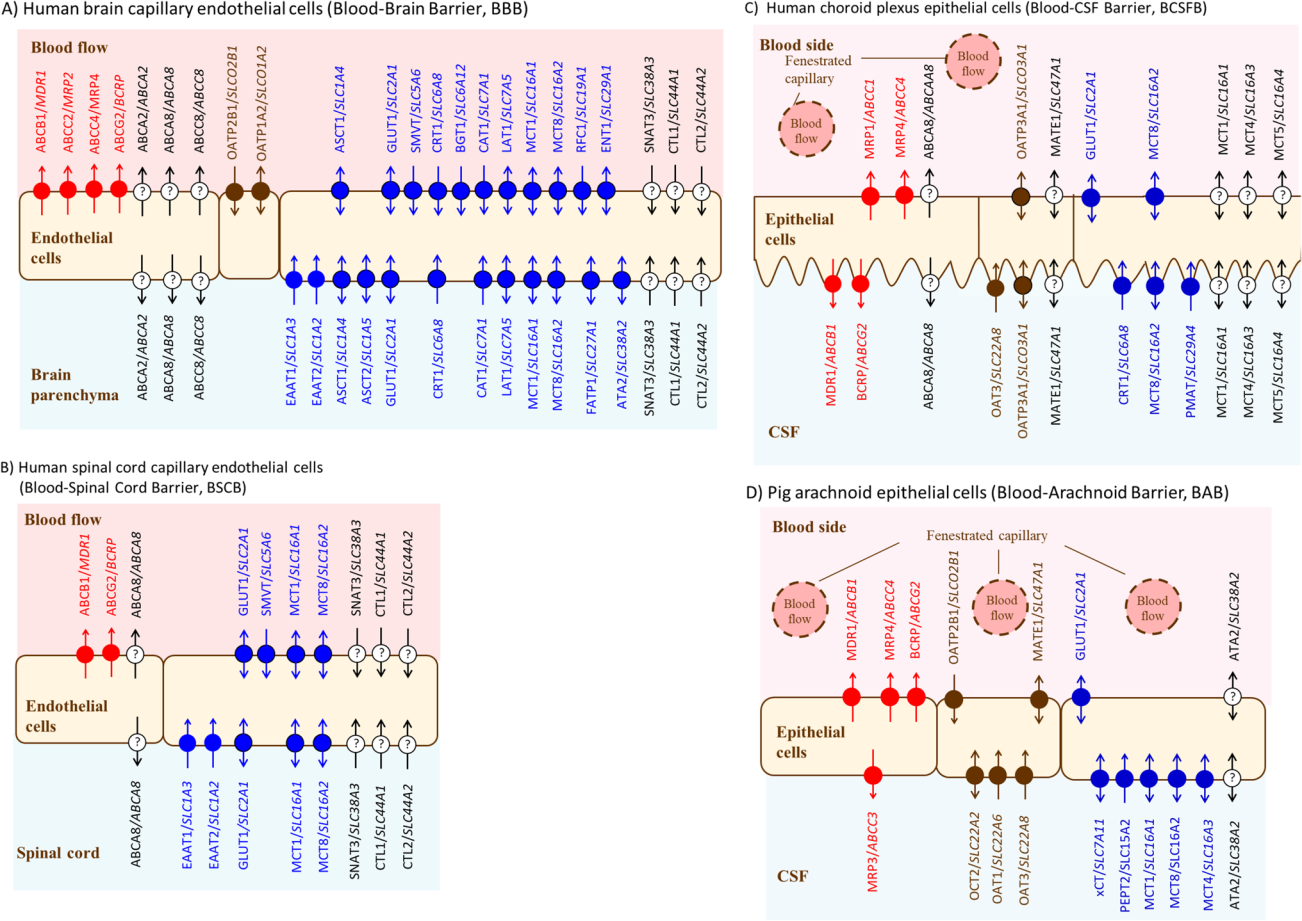
Transporter protein localization in the CNS barriers. The expression and localizations of transporters are taken from previous reports (5, 8, 31, 35–47). Symbols with a question mark (?) indicate transporters that have not been confirmed the localization.

Breast cancer resistant protein, BCRP/*ABCG2* is a well-known drug efflux transporter (28, 29). The transporter protein expression of BCRP in the brain capillary endothelial cells in the dog is 45.2 ± 10.8 (fmol/μg protein) (30), which is 5.6-fold greater than in humans (8.14 ± 2.26 (fmol/μg protein)) (9) and 9.1-fold greater than in rats (4.95 ± 0.32 (fmol/μg protein)) (6). In the choroid plexus epithelial cells, BCRP protein is located in the plasma membrane facing CSF in mice (31) (Figs. 1 and 2C). BCRP protein has not been detected in the dog choroid plexus, indicating no functional contribution of BCRP at the BCSFB in the dog (30). Accordingly, the dog is an appropriate animal model to examine the contribution of transport function at the BBB for the drug distribution in the CSF. K_p,uu,brain_ of daidzein, a selective substrate of BCRP, has been reported to be 0.115 ± 0.006 (30), which is affected by the BCRP efflux transport at the BBB. Interestingly, K_p,uu,csf_ of daidzein was in turn 0.792 ± 0.14 and similar to that of antipyrine (0.918 ± 0.085), which is a representative drug that permeates through the plasma membrane by passive diffusion. Although several reports are showing a fairly good correlation between K_p,uu,brain_ and K_p,uu,csf_ (32–34), the results shown in the dog (30) prove clearly that drug concentration in the CSF does not reflect the BBB transport function.

The volume of CSF (82%) is located in the subarachnoid space (Fig. 1). Therefore, the substrate concentration in the CSF is significantly affected by the transport function of BAB (35, 48) (Figs. 1 and 2D). If the CSF were collected from the cerebroventricular space in a smaller volume than that of the space, the drug concentration would be mainly reflected by the transport function of the BCSFB (Figs. 1 and 2C). If the CSF were collected from the lumbar spine, the concentration would be reflected by the transport function of the BCSFB (Fig. 2C), BAB (Fig. 2D), and BSCB (Figs. 1 and 2B).

Regarding the drug concentration in the CSF, the water flow in the brain parenchymal tissue must also be considered. CSF, which is produced primarily by the choroid plexus in the ventricles (23, 49), flows from the subarachnoid space to the arachnoid villi, and returns to the circulating blood from the superior sagittal sinus. Recently, two theories have been proposed, i.e., 1) the perivascular pathway theory (50, 51), in which “a part of CSF has a pathway that flows in the direction opposite to the blood flow around the brain side of the capillary endothelial cells of the brain parenchymal tissue”, and 2) the glymphatic pathway hypothesis (52, 53), in which “there is a partial pathway of CSF flow around the brain side of arterial vascular endothelial cells, that flows through the interstitial fluid of brain cells via glial cells, flows around the brain side of venous capillary endothelial cells and migrates to the subarachnoid space”. Although a common to these both theories is that “a flow exists near the brain side of brain capillary endothelial cells”, further studies will be necessary to verify “the significance of the interstitial fluid flow contributing to the substrate flow in the brain parenchymal tissue”(54, 55).

## Comparison of the Transporter Protein Expression between the Blood-Brain Barrier (BBB) and the Blood-Spinal Cord Barrier (BSCB)

### Transporter Protein Expression per Wet Tissue Weight (pmol/g) Is a Valuable Unit for the Understanding of Transport Capacity and Parameters for Pharmacokinetic (PK) Simulation

**Scheme 1:**
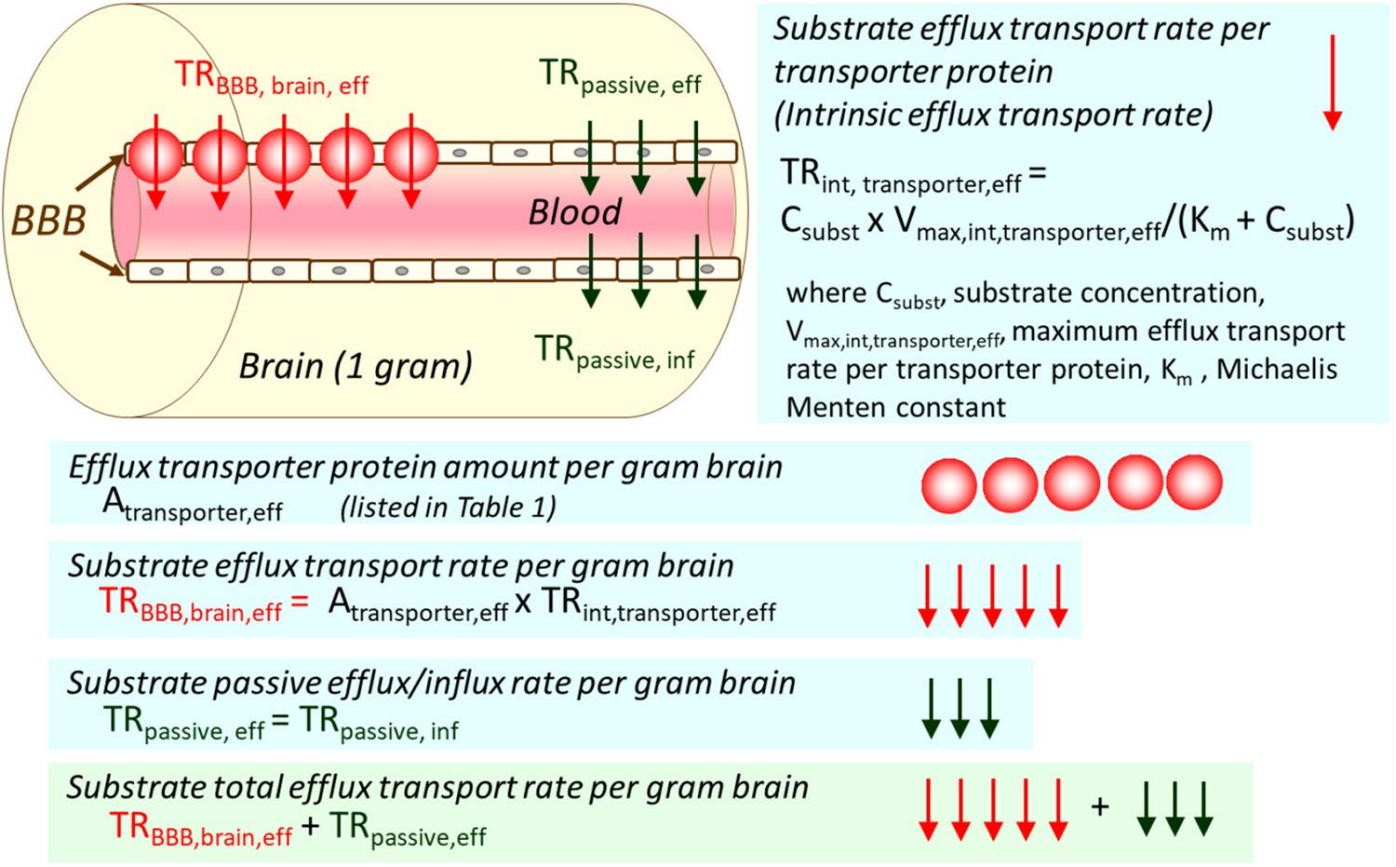
Prediction of *in vivo* BBB transport rate per gram brain from the BBB transporter protein amount per gram brain (A_transporter_) and the transport rate per transporter protein (TR_int, transporter_).

For the prediction of *in vivo* BBB transport rate from *in vitro* uptake study, it is important to use a unit of transporter protein expression per wet tissue weight (pmol/g). Previously, transporter protein expression has been reported with a unit of transporter protein per protein amount of the sample analyzed, such as whole cell lysate of the isolated brain capillaries, crude membrane fraction, or plasma membrane fraction (fmol/μg protein). We have recently established a method to convert the unit from (fmol/μg protein) to (pmol/g tissue weight) (5, 36). Briefly, the recovery of sample purification was determined by the quantification of the marker protein selectively expressing in the sample to be quantified, e.g.*,* GLUT1/*SLC2A1* for a vascular marker of the brain (5) both in the sample and whole cell lysate of the organ, such as brain or spinal cord. One of the advantages of this unit conversion is to counterbalance the purity of the sample preparation, and thereby the inter-laboratory differences of the determined membrane protein amount can be minimized. Moreover, it is useful to compare the transport capacity of the respective transporter in different barriers by assuming the transporter protein expression per grams of tissue weight correlates with the maximum transport rate of the transporter. More importantly, it enables us to predict *in vivo* BBB transport rate per grams of tissue (μL/(min x g tissue) by multiplying the transporter protein expression per grams of wet tissue weight (pmol/g) (Table I) and the intrinsic transport rate, i.e.*,* the transport rate per transporter protein expression in the cells (*in vitro*; μL/(min x pmol)). Scheme 1 illustrates the outline of how to predict the *in vivo* BBB transport rate per gram brain from the transporter protein expression per gram brain and the transport rate per transporter protein. In the pharmacokinetic (PK) modeling and simulation, it is noteworthy that the passive diffusion process also needs to be incorporated in the PK modeling and simulation (Scheme 1).

**Table I:** Comparison of Transporter Protein Expression Between the Blood-Brain Barrier (BBB) and the Blood-Spinal Cord Barrier (BSCB) in Human

Transporter	Protein expression (pmol/g wet tissue weight)		
	Isolated capillaries from brain cortex	Isolated capillaries from spinal cord	Ratio
	BBB	BSCB	(BBB /BSCB)
Efflux transporter			
MDR1/*ABCB1*	9.05 ± 5.15	1.93 ± 1.10	4.69
BCRP/*ABCG2*	7.47 ± 3.12	2.11 ± 1.58	3.53
ABCA8/*ABCA8*	1.55 ± 0.48	1.54 ± 0.95	1.00
Thyroid hormone transporter			
MCT8/ *SLC16A2*	6.19 ± 2.41	6.06 ± 2.68	1.02
Energy source transporter			
GLUT1/ *SLC2A1*	77.4 ± 31.5	20.6 ± 10.2	3.76
MCT1/ *SLC16A1*	2.85 ± 1.06	0.892 ± 0.154	3.19
Amino acid transporter			
EAAT2/ *SLC1A2*	6.46 ± 3.20	2.10 ± 2.60	3.07
EAAT1/ *SLC1A3*	24.4 ± 10.7	13.2 ± 6.0	1.85
4F2hc/ *SLC3A2*	3.79 ± 2.26	1.51 ± 0.65	2.50
SNAT3/ *SLC38A3*	2.14 ± 0.91	0.407	5.27
Vitamin transporter			
SMVT/ *SLC5A6*	19.1 ± 7.8	18.6 ± 8.5	1.02
Choline transporter			
CTL1/ *SLC44A1*	5.59 ± 2.81	6.31 ± 3.05	0.89
CTL2/ *SLC44A2*	12.7 ± 4.9	7.37 ± 3.21	1.72

Values were cited from supplemental Table S2 in reference (5). GLUT 1 was used as a vascular endothelial cell marker. The protein expression level of GLUT1 was used for the unit conversion from (units: fmol/μg protein) to (units: pmol/g tissue). The average of the frontal cortex and temporal/parietal cortex region of 3 donors, and that of the spinal cord of 4 donors are shown with the standard deviation (SD) in the table

### The BBB and the BSCB Have Similar Transport Functions but Different Transport Capacity

Table I lists the transporter protein expression per grams of wet tissue weight (pmol/g) in the BBB and BSCB for humans (5). Figure 2A and B illustrate the localization of transporter proteins in the BBB and BSCB, respectively. One of the most important findings is that the multidrug resistance protein 1 (MDR1/*ABCB1*) protein expression in the BBB is 4.69-fold greater than that of the BSCB in humans. The BCRP protein expression in the BBB is 3.53-fold greater than that of BSCB. These notable differences suggest that substrates of MDR1 and BCRP may distribute in the spinal cord remarkably higher than those of the brain. As summarized in Table I, the BBB transporter protein expression of glucose (GLUT1), monocarboxylic acid (MCT1/*SLC16A1*), amino acid (EAAT1/*SLC1A3*, SNAT3/ *SLC38A3*) are 3-fold greater than those of the BSCB. Interestingly, it has been reported that the BBB transport rate of ^18^F-FDG (2-deoxy-2-[^18^F]fluoroglucose), a substrate of GLUT1, is 4.8-fold greater than that of the BSCB (56, 57), suggesting that the differential GLUT1 protein expression between BBB and BSCB generates the differential *in vivo* transport rate between BBB and BSCB. Although the drug diffusion in the spinal cord is limited, the blood-arachnoid barrier (BAB) surrounding the CSF would participate in decreasing the drug concentration in the CSF by pumping out the substrates of MDR1 and BCRP to the circulating blood (Figs. 1 and 2D).

### Transporter Protein Expression per Surface Area of the Barrier (fmol/cm^2^) Is an Important Unit for the Prediction of K_p,uu,brain_

Considering a drug of MDR1 substrate, an efflux transport activity/rate per 1 fmol of MDR1 (μL/(min x fmol)) indicates TA_MDR1_, a passive transport rate per cm^2^ at the luminal and abluminal plasma membrane (μL/(min x cm^2^)) of the brain capillaries indicates P_passive_, MDR1 protein expression level per surface area of the brain capillaries (fmol/cm^2^) indicates PEL_MDR1_. Assuming no additional carrier-mediated transport is participating the BBB transport, K_p,uu,brain_ can be described as (5):

3 $$\begin{aligned}{\displaystyle \begin{array}{l}{\mathrm{K}}_{\mathrm{p},\mathrm{uu},\mathrm{brain}}=\frac{P_{passive}}{P_{passive}+{\mathrm{PEL}}_{\mathrm{MDR}1}\times {\mathrm{TA}}_{\mathrm{MDR}1}}\\ {}\kern5.75em =\frac{1}{1+{\mathrm{PEL}}_{\mathrm{MDR}1}\times \left({\mathrm{TA}}_{\mathrm{MDR}1} \left/ {P}_{passive}\right.\right)}\end{array}}\end{aligned}$$

Equation 3 indicates that K_p,uu,brain_ is predicted by the reciprocal value of 1 plus the ratio of transport rate per surface area at the luminal plasma membrane between the MDR1 mediated efflux transport and the passive diffusion. In other words, the ratio of transport rate per surface area between MDR1 mediated efflux rate and the passive diffusion rate explains the unbound drug concentration gradient generated by the efflux transport function of MDR1 at the luminal membrane of the brain capillaries. Accordingly, the unit of transporter protein expression per surface area is important for the comparison of K_p,uu_ in the different barrier regions, different animal species, and different disease conditions.

### MDR1 Protein Expression per Surface Area in the Human BBB and BSCB Is 10- and 30-Times Smaller than that of Rats, Respectively

Table II lists the protein expression level/amount (PEL) of MDR1, BCRP, and bile acid and sterol efflux transporter A8 (*ABCA8*)(58) with the unit of transporter protein expression per surface area of the capillary endothelial cells in the cerebral cortex, cerebral white matter, and thoracic spinal cord in humans (fmol/cm^2^) (5) together with those of cerebrum and spinal cord in rats. MDR1 and BCRP protein expressions in cerebral white matter are approximately 30% smaller than those of the cerebral cortex, suggesting a small regional difference of the BBB efflux transporters in humans. Interestingly, MDR1 protein expression in the human cerebral cortex was 10-times smaller than that of the rat cerebrum (Table II). In addition, BCRP protein expression in the human cerebral cortex is 5-times smaller than that of the rat cerebrum (Table II).

**Table II:** Comparison of ABC Transporter Protein Expression per Surface Area of the Blood-Brain Barrier (BBB) and the Blood-Spinal Cord Barrier (BSCB) Between Humans and Rats

	Protein expression (PEL)	(fmol/cm^2^ surface area of the barrier)			
	ABCA8/*ABCA8*	MDR1/*ABCB1*		BCRP/*ABCG2*	
	humans	humans	rats	humans	rats
Blood-brain barrier (BBB)					
Cerebral cortex	8.62 ± 2.66	50.3 ± 28.6		41.5 ± 17.3	
Cerebral white matter	7.54 ± 1.10	33.1 ± 15.6		30.8 ± 6.3	
Cerebrum			497 ± 15		217 ± 2
Blood-spinal cord barrier (BSCB)					
Thoracic spinal cord	10.9 ± 6.7	13.7 ± 7.8		15.0 ± 11.2	
Spinal cord			398 ± 26		252 ± 3

Values were cited from supplemental Table S3 in reference (5). For the unit conversion from (pmol/g wet tissue weight) to (fmol/cm^2^ surface area), the following values were used: 180 cm^2^/g wet tissue for cortex in humans (59), 100 cm2/g tissue for white matter in humans (59), and 141 cm^2^/g tissue for the spinal cord in humans (5), 140 cm^2^/g tissue weight for the whole cerebrum in rats (60), and 159 cm^2^/g wet tissue for the spinal cord in rats (61), respectively. The average of the frontal cortex and temporal/parietal cortex region of 3 donors, and that of the spinal cord of 4 donors are shown with the standard deviation (SD) for humans. Capillaries were isolated from the pooled rat brains (n = 9) and spinal cord (n = 18). The averages are shown with the standard error of the mean (S.E.M) for rats

MDR1 protein expression level per surface area (PEL_MDR1_) in the human spinal cord is 35% of that of the human cerebral cortex and white matter (Table II). BCRP protein expression per surface area in the human spinal cord is in turn 43% of that of the human cerebral cortex and white matter (Table II). Interestingly, MDR1 protein expression per surface area in the rat spinal cord is 80% of that of the rat cerebrum (Table II). On the contrary, BCRP protein expression per surface area in the rat spinal cord is 120% of that of the rat cerebrum (Table II). Based on these protein expressions of MDR1 and BCRP in BBB and BSCB (Table II), it can be predicted that K_p,uu_ of MDR1 and BCRP substrates in the spinal cord would be similar to those of the brain in rats. MDR1 and BCRP protein expressions per surface area in humans are 30- and 17-times smaller than those of rats, respectively, suggesting that K_p,uu_ of MDR1 and BCRP substrates in the human spinal cord can be 30-fold and 17-fold higher than those of rats. When extrapolating pharmacological and toxicological effects of drugs transported by MDR1 and BCRP to the spinal cord, it would be important to take into consideration of these remarkably lower expression profiles of MDR1 and BCRP in the spinal cord compared to the rats.

## Prediction of the Unbound Drug Concentration Ratio Between Brain And Blood (K_**p,uu,brain**_)

### K_p,uu,brain_ Can Be Predicted from the BBB MDR1 Protein Expression and Intrinsic Efflux Activity of MDR1

**Fig. 3:**
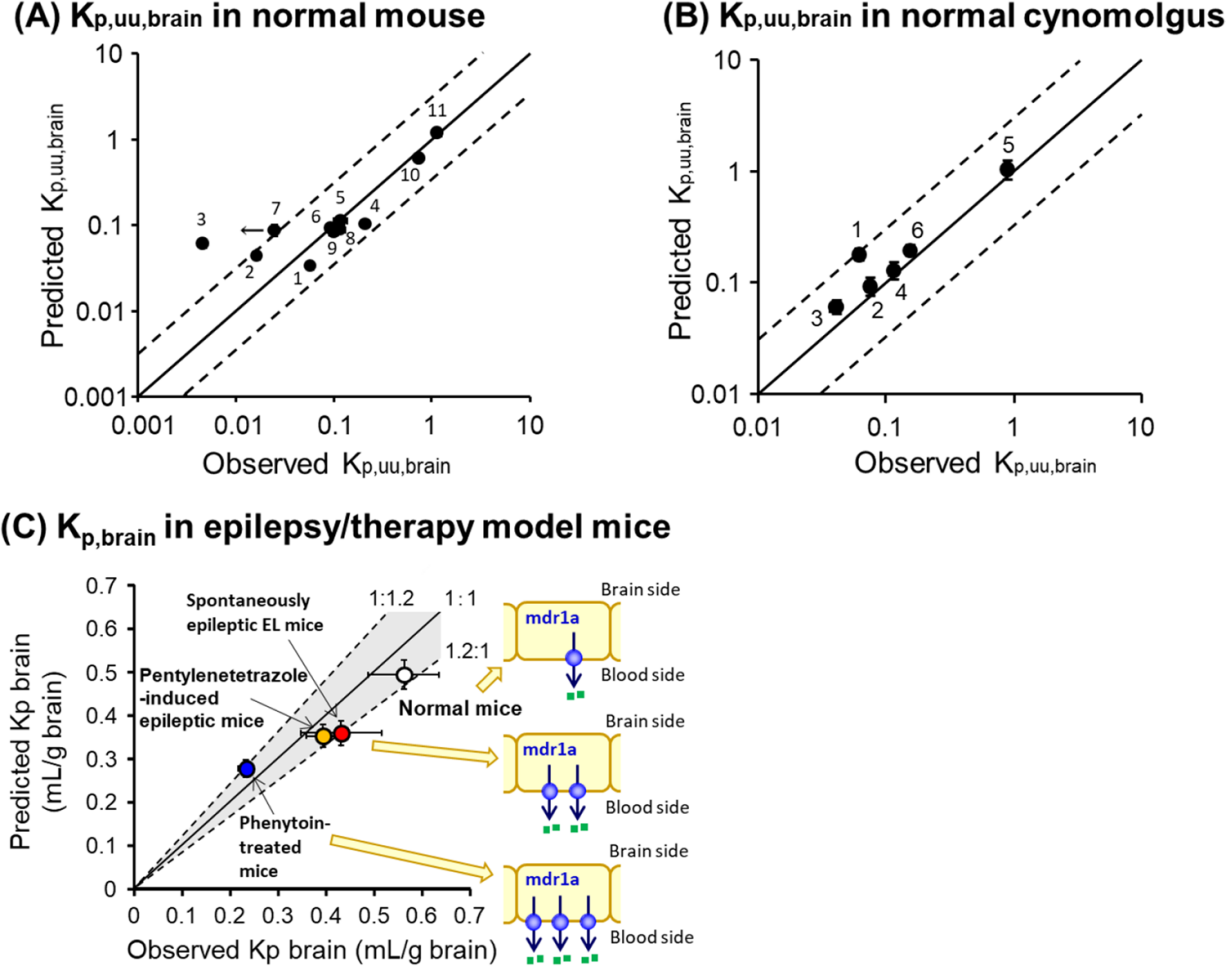
Pharmacoproteomics-based prediction of drug concentration ratio of brain and plasma (K_p,uu,brain_ and K_p,brain_) for mdr1a/MDR1 substrates from *in vitro* experiments. (**A**) Validation using normal mice. 1, quinidine; 2, loperamide; 3, digoxin; 4, risperidone; 5, indinavir; 6, dexamethasone; 7, vinblastine; 8, paclitaxel; 9, verapamil; 10, loratadine; 11, diazepam. The observed K_p,uu,brain_ of vinblastine was less than 0.0248. (**B**) Validation using normal cynomolgus. 1, indinavir; 2, quinidine; 3, loperamide; 4, paclitaxel; 5, diazepam; 6, verapamil. (**C**) Validation using epilepsy/therapy model mice. Data were cited from (13–15) and modified.

Protein expression levels of the BBB transporters differ notably in humans and rodents (Table II) (5). Large quantitative differences are also observed in the transport molecular mechanisms between *in vitro* cultured human brain capillary endothelial cells and *in vivo* BBB (62). These results indicate that conventional animal experiments and *in vitro* analyses cannot predict the drug permeability across the BBB *in vivo* in humans. Therefore, we have developed a mathematical model to predict the drug transport across the BBB; by integrating the MDR1 protein expression levels in brain microvessels and the drug efflux transport activity per mdr1a/MDR1 protein ([(*in vitro* MDR1 efflux ratio)-1]/[MDR1 protein expression levels in MDR1-transfected cell monolayer]) the *in vivo* mdr1a/MDR1 efflux activity at the BBB can be predicted from *in vitro* experiments (13). We have also demonstrated that it is possible to predict the concentration of unbound drugs in the brain using a mouse model based on the following theory (Eq. 4) (Fig. 3A) (13):

4 $${\mathrm{K}}_{\mathrm{p},\mathrm{uu},\mathrm{brain}}=\frac{1}{1+\left(\left( in\ vitro\ \mathrm{MDR}1\ \mathrm{efflux}\ \mathrm{ratio}\right)-1\right)\times \frac{\mathrm{MDR}1\ \mathrm{protein}\ \mathrm{expression}\ \mathrm{level}\ \mathrm{in}\ \mathrm{brain}\ \mathrm{microvessels}}{\mathrm{MDR}1\ \mathrm{protein}\ \mathrm{expression}\ \mathrm{level}\ \mathrm{in}\ \mathrm{MDR}1-\mathrm{transfected}\ \mathrm{cell}\ \mathrm{monolayer}}}$$

Unlike the mice, the protein expression level of MDR1 at the BBB in cynomolgus is quite similar to that in humans (7). Therefore, using the cynomolgus model, we have also demonstrated that the predictive theory above can be used to predict the concentration of unbound drugs in the brain in cynomolgus, a non-human primate (Fig. 3B) (15). Furthermore, the transport functions at the BBB quantitatively change in CNS diseases. Using epilepsy and phenytoin-treated mice models, we have demonstrated that it is possible to predict the changes in the brain concentration of mdr1a/MDR1 substrate in response to pathology and treatment (Fig. 3C) (14). It is therefore expected that our prediction method will apply to patients with CNS diseases. These results have opened the way to quantitative prediction of the unbound drug concentration of transporter substrates in the brain, and will contribute greatly to the development of the new research field of “*Pharmacoproteomics*”, which combines pharmacokinetics and proteomics (63).

### K_p,uu,brain_ May Be Predicted for Humans from Rats

In order to evaluate the effect of the significant inter-species difference of the BBB efflux transporter protein expression (Table II), Eq. 5 can be derived from Eq. 3,

5 $$\frac{{\mathrm{TA}}_{in\ vivo,\kern0.5em rat}}{P_{passive,\kern0.5em in\ vivo,\kern0.5em rat}}=\frac{\left(\left(\frac{1}{{\mathrm{K}}_{p, uu, brain, rat}}\right)-1\right)}{{\mathrm{PEL}}_{rat}}$$

Considering MDR1 substrates for the brain distribution in rats, TA_vivo,MDR1_/P_passive,vivo,MDR1_ can be estimated by Eq. 5 with changing K_p,uu,brain,MDR1,rat_ from 1.00 × 10^−4^ to 9.90 × 10^−1^ with PEL_MDR1,rat_ of rat cerebrum (497 ± 15 fmol/cm^2^) (Table S1). The ratio of TA_vivo_/P_passive,vivo_ between humans and rats can be defined as RTA_human/rat_ by Eq. 6:

6 $${\mathrm{RTA}}_{\mathrm{human}/\mathrm{rat}}=\frac{{{\mathrm{TA}}_{in\ vivo, human}} \left/ {{P}_{passive, in\ vivo, human}}\right.}{{{\mathrm{TA}}_{in\ vivo, rat}} \left/ {{P}_{passive, in\ vivo, rat}}\right.}$$

Equation 7 can be derived from Eq.3 for the prediction of K_p,uu,brain,human_ in human:

7 $${\mathrm{K}}_{\mathrm{p},\mathrm{uu},\mathrm{brain},\mathrm{human}}=\frac{1}{1+{\mathrm{PEL}}_{human}\times \left({{\mathrm{TA}}_{in\ vivo, rat}} \left/ {{P}_{passive, in\ vivo, rat}}\right.\right)\times {\mathrm{RTA}}_{human/ rat}}$$

**Fig. 4:**
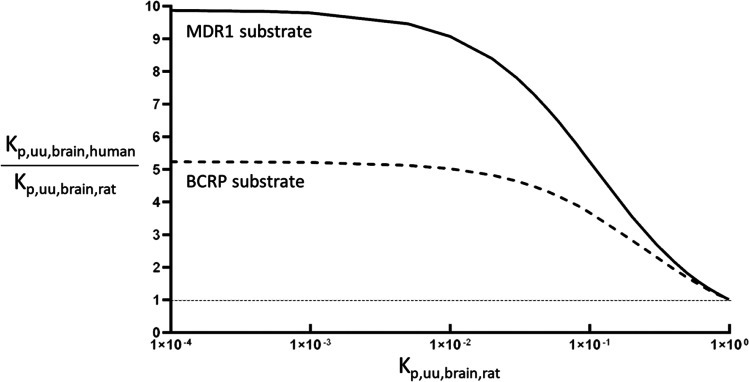
Effect of species difference of MDR1 and BCRP protein expression in the BBB to the ratio of K_p,uu,brain,human_/K_p,uu,brain,rat_. K_p,uu,human_ was estimated from K_p,uu,rat_ and K_p,uu,human_/K_p,uu,rat_ was plotted for K_p,uu,rat_. MDR1 substrate (solid line): changing K_p,uu,brain,MDR1,rat_ from 1.00 × 10^−4^ to 9.90 × 10^−1^ with PEL_MDR1,rat_ of rat cerebrum (497 fmol/cm^2^), TA_MDR1, rat, vivo_/P_passive,rat,vivo_ was estimated by Eq. 5. Assuming RTA_MDR1,human/rat_ is 1.0, K_p,uu,brain,MDR1,human_ was predicted by Eq. 7 with PEL_MDR1,human_ of human cerebral cortex (50.3 fmol/cm^2^). BCRP substrate (broken line): changing K_p,uu,brain,BCRP,rat_ from 1.00 × 10^−4^ to 9.90 × 10^−1^ with PEL_BCRP,rat_ of rat cerebrum (217 fmol/cm^2^), TA_BCRP, rat, vivo_/P_passive,rat,vivo_ was estimated by Eq. 5. Assuming RTA_BCRP, human/rat_ is 1.0, K_p,uu,brain,BCRP,human_ was predicted by Eq. 7 with PEL_BCRP,human_ of human cerebral cortex (41.5 fmol/cm^2^). Dotted line indicates K_p,uu,human_/K_p,uu,rat_ is 1.0, i.e., no species difference.

Assuming there is no species difference of MDR1 efflux activity per MDR1 protein, i.e., RTA_MDR1, human/rat_ = 1.0, K_p,uu,brain,human_ can be predicted by Eq. 7 with TA_vivo,MDR1_/P_passive,vivo,MDR1_ and PEL_MDR1,human_ of human cerebral cortex (Table II, 50.3 ± 28.6 fmol/cm^2^). If K_p,uu,brain,MDR1_ in rats is smaller than 1.00 × 10^−2^, it can be predicted that K_p,uu,brain,MDR1_ in humans is 9-fold greater than that of rats. If K_p,uu,brain,MDR1_ in rats is in the range between 1.00 × 10^−2^ and 1.00 × 10^−1^, K_p,uu,brain,MDR1_ in humans can be 5–9 -fold greater than that of rats. Interestingly, it has been reported that K_p,uu,brain_ of verapamil, a substrate of MDR1, is 0.24 in humans (64) and 0.0786 in rats (65), showing 3.1-fold higher K_p,uu,brain_ of verapamil in humans than that of rats. However, the predicted result has 5.8-fold greater K_p,uu,brain,MDR1_ in humans than that of rats, which is a twice greater value than the observed value. However, a possibility that RTA_verapamil,MDR1,human/rat_ might be greater than 1.0, e.g.*,* 2.68, cannot be excluded. The ratio of K_p,uu,brain,MDR1,human_/K_p,uu,brain,MDR1,rat_ was also determined and plotted for K_p,uu,brain,MDR1,rat_ (Fig. 4), which would be a useful parameter for the prediction of K_p,uu,brain,MDR1,human_ from rats to human as a conventional scale-up factor (see supplemental Table S1). If RTA_MDR1,human/rat_ could be determined by the *in vitro* transport study with quantification of MDR1 in rats and humans, the reliability of the prediction of K_p,uu,brain,MDR1,human_ by Eq. 7 would be increased. Similar to MDR1, effects of a 5-times difference of BCRP protein expression in the BBB on the inter-species difference of K_p,uu,brain_ between humans and rats are also simulated and illustrated in Fig. 4 (supplemental Table S2). Further studies would be necessary to validate the predicted results in Fig. 4 by measuring K_p,uu,brain_ in humans. Nevertheless, it is encouraged to re-analyze the data using Eq. 7 by measuring the protein expression of MDR1 in the *in vitro* culture system used for the drug screening.

## Transporter Protein Expression in the Blood-Cerebrospinal Fluid Barrier (BCSFB)

Table III lists the transporter protein expression level in the fourth ventricular choroid plexus in humans (66), i.e.*,* the blood-CSF barrier (BCSFB). Figure 2C illustrates the localization of transporter proteins in the BCSFB. Similar to that of the BBB shown in Table I, GLUT1 protein expression is highest, 46.7 ± 1.1 fmol/μg protein. Interestingly, protein expression of an organic cation transporter, multidrug and toxin extrusion 1 (MATE1/*SCL47A1*), organic anion transporter 3,(OAT3/*SLC22A8*) and organic anion transporting polypeptide 3A1 (OATP3A1/*SLCO3A1*) are 8.61 ± 0.63 fmol/μg protein, 1.87 ± 0.12 fmol/μg protein, 0.641 ± 0.119 fmol/μg protein, respectively (Table III), while these transporter proteins were not identified in the human BBB.

**Table III:** Transporter Protein Expression in the Blood-Cerebrospinal Fluid Barrier (BCSFB) in Human

Transporter	Protein expression (fmol/μg protein) (mean ± SD)
Efflux transporter	
MDR1/*ABCB1*	2.10 ± 0.17
BCRP/*ABCG2*	0.706 ± 0.053
MRP1/*ABCC1*	1.36 ± 0.11
MRP4/*ABCC4*	0.818 ± 0.142
*ABCA8/ABCA8*	1.52 ± 0.27
Organic anion transporter	
OAT3/*SLC22A8*	1.87 ± 0.12
OATP3A1/*SLC21A11/SLCO3A1*	0.641 ± 0.119
Organic cation transporter	
MATE1/*SLC47A1*	8.61 ± 0.63
Thyroid hormone transporter	
MCT8/*SLC16A2*	1.65 ± 0.16
Energy source transporter	
GLUT1*/SLC2A1*	46.7 ± 1.1
GLUT5/*SLC2A5*	1.24 ± 0.19
GLUT3,14/*SLC2A3, SLC2A14*	0.472 ± 0.035
MCT1/*SLC16A1*	3.47 ± 0.26
MCT4/*SLC16A3*	0.382 ± 0.078
MCT5/*SLC16A4*	0.685 ± 0.124
Amino acid transporter	
EAAT1/*SLC1A3*	5.04 ± 0.18
CAT1/*SLC7A1*	1.22 ± 0.15
4F2hc/*SLC3A2*	1.42 ± 0.28
Folic acid transporter	
RFC1/*SLC19A1*	3.68 ± 0.09
PCFT/*SLC46A1*	1.78 ± 0.17
Creatine transporter	
CRT1/*SLC6A8*	0.450 ± 0.138
Nucleoside transporter	
ENT1/SLC29A1	2.49 ± 0.12
Monoamine transporter	
PMAT/SLC29A4	0.288 ± 0.041

Values were obtained from Table I published in the reference (66). Choroid plexus from the fourth ventricle of 92 years old male was quantified by LC-MS/MS as 3 replicates. S.D. value indicates a variation of the analysis

Relatively abundant protein expression of folic acid transporters, PCFT/*SLC46A1* (proton-coupled folate transporter) and RFC1/*SLC19A1* (reduced folate carrier 1) has also been found in the human BCSFB. PCFT has a strong affinity for folic acid transport at about 1 μM K_m_ (67), while RFC1 has a very weak affinity for folic acid transport, at about 100 μM K_m_ (68). Methotrexate (MTX) used in MTX-leucovorin rescue therapy is a structural analog of folic acid, and its K_m_ values for both transport carriers are 2 to 10 μM (67, 68). It is considered that both transport carriers contribute to the transport of MTX from the circulating blood to the CSF. A correlation between the risk of developing adverse reactions during the treatment of acute leukemia with high-dose MTX and the RFC1 gene (*SLC19A1*) polymorphism has been already reported (69, 70). Dosage design based on kinetic evaluation, including the contribution of RFC1 and PCFT in the transport of MTX in the blood to the CSF, may be an important subject to be clarified for the future.

MDR1 and BCRP are also expressed in the BCSFB (Table III), while both are localized in the plasma membrane of choroid plexus epithelial cells facing the CSF (31, 37) (Fig. 2C). In the BBB, both transporters are preventing substrate entry from the circulating blood to the brain, whereas the direction of both efflux transporters in the BCSFB is the opposite of the BBB, i.e., from epithelial cells to the CSF (Fig. 2A). There is a report supporting the differential *in vivo* contribution of MDR1 and BCRP to the brain parenchymal and the CSF (71). The brain distribution of topotecan, a substrate of MDR1 and BCRP was significantly increased after the systemic administration to the knockout mice with the *Mdr1a, Mdr1b,* and *Bcrp1* genes, whereas the intraventricular CSF topotecan concentration measured by microdialysis decreased significantly in the knockout mice with the *Mdr1a, Mdr1b,* and *Bcrp1,* genes (71). Considering the barrier function of the choroid plexus, i.e.*,* preventing xenobiotics entry from the blood in the CSF, the ability of MDR1 and BCRP to transport xenobiotics in the blood towards the CSF across the BCSFB may not be a rational physiological route. Considering endogenous substrates to be transported by MDR1, e.g., glucocorticoid (72), estrone and estriol (73), endomorphin-1 (74), it can be hypothesized that MDR1 and BCRP in the choroid plexus play a role in supplying endogenous substances to the circumventricular organ and/or any region of subarachnoid space facing with CSF (Fig. 1), which may be necessary for any CNS function in the region. Thus, there will be a significant risk caused by xenobiotics transport by MDR1 and BCRP from the circulating blood to the CSF across the BCSFB (Fig. 2C). However, the CSF xenobiotics concentration in subarachnoid space would be decreased effectively by the efflux transport function of the blood-arachnoid barrier (BAB) (Fig. 2D) (35, 36, 48). Therefore, when analyzing the drug concentrations in the CSF, it should be noted that the small samples collected from the cisternal magna reflect the influx function of MDR1 and BCRP at the BCSFB, whereas the samples collected from the lumber corresponds the efflux function of MDR1 and BCRP at the BAB, BSCB, and the influx function of MDR1 and BCRP at BCSFB.

## Characterization of Transport Function of the Blood-Arachnoid Barrier (BAB)

### The BAB Plays a Major Clearance Pathway of Organic Anions from CSF

**Fig. 5:**
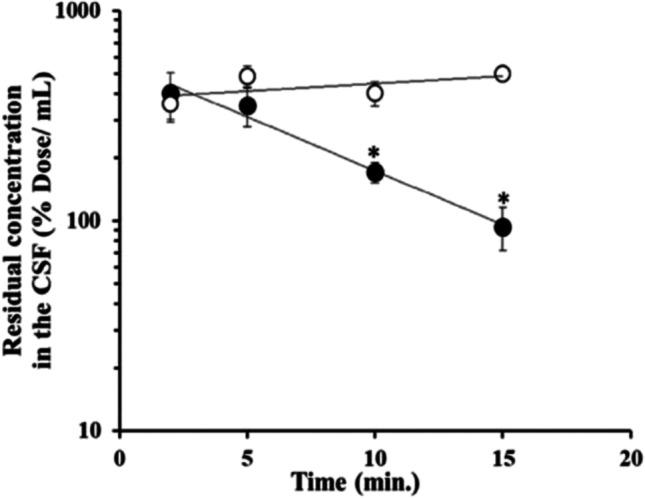
The concentration of *para*-aminohippuric acid (PAH) (●) and FITC-inulin (○) in the cisterna CSF *versus* time profile after intracisterna magna (*i.c.m.*) administration. FITC-inulin was used as a reference for CSF bulk flow turnover and passive diffusion into the spinal cord. Each point represents the mean ± SEM (n = 6–10). The values are expressed as the percentage of the dose remaining per milliliter of CSF. An asterisk (^∗^) denotes values of of PAH and FITC-inulin (% dose/mL) that were significantly different (p < 0.01). The figure was reprinted (adapted) with permission from reference (48). Copyright 2022 American Chemical Society.

It has been believed for several decades that arachnoid epithelial cells do not permeate water-soluble substances (49). Yasuda *et al*. have reported that MDR1 is expressed at the apical membrane of arachnoid epithelial cells of mouse, monkey, and human leptomeninges (38). Several transporter genes were also identified in the arachnoid epithelial cells, e.g., Mdr1a*/Abcc1a,* Bcrp*/Abcg2*, Oat1*/Slc22a6,* Oat3/*Slc22a8,* Mrp1*/Abcc1,* Mrp4/*Abcc4* in mouse and MDR1*/ABCB1,* BCRP*/ABCG2,* OAT1*/SLC22A6,* OAT3*/SLC22A8,* OCT1*/SLC22A2* in human (38). In order to prove that the arachnoid epithelial cells have substantial *in vivo* transport function, the intracisternal magna (*i.c.m.*) administration, which can prevent the transport function of choroid plexus in the ventricular space, has been employed. As shown in Fig. 5 (48), a significant elimination of the *para*-aminohippuric acid (PAH) concentration in the cisterna CSF was demonstrated with the elimination clearance of 26.5 μL/min, which was remarkably greater than the CSF bulk flow rate (2 μL/min) (75). Comparing the elimination clearance of PAH with the CSF volume in rats, 250 μL (76), the turnover rate is 9 min, suggesting major *in vivo* transport activity from the CSF in the subarachnoid space. Furthermore, leptomeninges containing arachnoid epithelial cells express Oat1 and Oat3 proteins; 2.73 ± 0.07 (fmol/μg protein) and 6.65 ± 0.20 (fmol/μg protein), respectively (48), and the elimination clearance of PAH was inhibited completely by ceftriaxone, an inhibitor of Oat1, and by 17% with cephalothin, an inhibitor of Oat3 (48, 66). Notably, Oat1 protein has been reported to be under the detection limit in the rat choroid plexus. These results suggested that Oat1 in the leptomeninges plays the major elimination pathway from the CSF in the subarachnoid space (48). Thus, this is the first report demonstrating the functional activity of the BAB for the avid clearance of organic anion in the subarachnoid CSF *in vivo.*

**Fig. 6:**
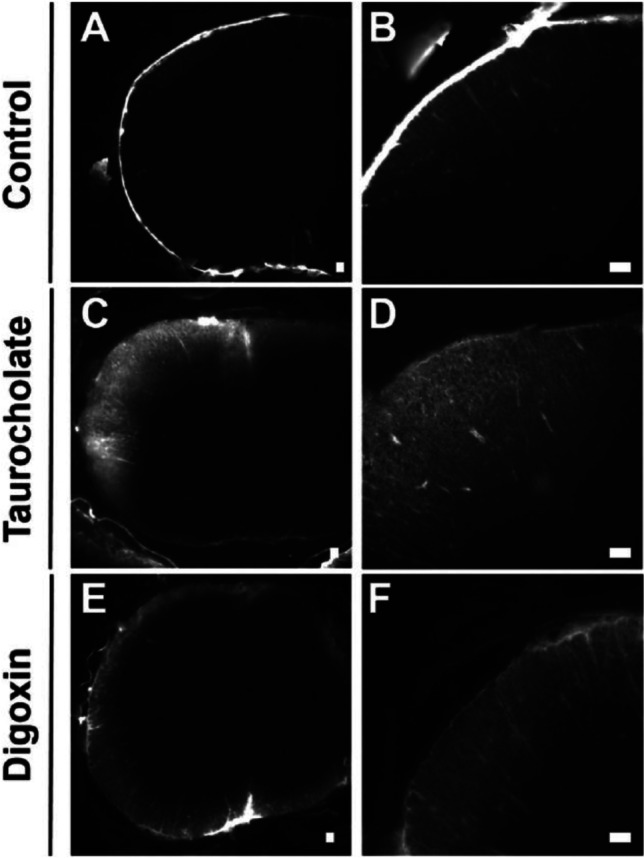
Distribution of SR-101 in the cervical spinal cord 20 min after intracisterna magna (*i.c.m.*) injection in rats. SR-101 was administered without inhibitor (**A, B**). The fluorescence signals of SR-101 were predominantly detected in the leptomeninges at the surface of the spinal cord. The fluorescence intensity of SR-101 was diminished in the leptomeninges and instead increased in the parenchyma of the spinal cord pre-administered with taurocholate (**C, D**) and digoxin (**E, F**). Scale bars: 300 μm. Subfigures B,D,F are enlarged spinal cord images of A,C,E, respectively. The figure was reprinted (adapted) with permission from reference (35). Copyright 2022 American Chemical Society.

Oatp1a4/*Slco1a4* protein expression has been quantified to be 8.86 ± 0.12 fmol/μg protein in the leptomeninges of rats, which is remarkably greater than that of Oat3. Although Oatp1c1/*Slco1c1* protein is 0.195 ± 0.018 fmol/μg protein in the leptomeninges of rats, the immunohistochemical analysis has indicated that Oatp1c1 protein is localized in blood vessels of the leptomeninges and not in the arachnoid matter (35). Figure 6 shows the distribution of SR-101, a representative substrate of organic anion, in the cervical spinal cord after *i.c.m* administration in rats. The fluorescence signals of SR-101 were predominantly detected in the leptomeninges at the surface of the spinal cord (Fig. 6 A, B), which is caused by the rapid uptake of SR-101 with Oatp1a4 in the leptomeninges. Although SR-101 is a substrate of Oatp1a4 and Oatp1c1 the contribution of Oatp1c1 for the uptake of SR-101 can be excluded due to the fact that Oatp1c1 protein was not detected in the arachnoid matter in the immunohistochemical analysis (35). Moreover, the fluorescence intensity of SR-101 was diminished in the leptomeninges and increasing in the parenchyma of the spinal cord when pre-administered with taurocholate (Fig. 6 C, D) and digoxin (Fig. 6E, F), a well-known broad-spectrum inhibitor of Oatps and a strong inhibitor of Oatp1a4, respectively (77, 78). Therefore, the results (Fig. 6) can be explained by the great inhibitory effect on the Oatp1a4-mediated uptake of SR-101 into the leptomeninges. Taking together, the arachnoid epithelial cells in the leptomeninges plays a crucial physiological and pharmacological role in CSF detoxification by restricting the distribution of organic anions to the spinal cord, and presumably brain parenchymal tissues (35).

### The Transporter Protein Amounts of the Blood-Arachnoid Barrier (BAB) Are Significantly Different from those of the Blood-Cerebrospinal Fluid Barrier (BCSFB)

The BAB surrounds 82% volume of the CSF in the subarachnoid space and 18% of the BCSFB in the ventricular space. The transporter protein expression amount in the total cerebral leptomeninges and those of total choroid plexus and the ratio of respective transporters between leptomeninges and choroid plexus were listed in Table IV (36). Each value reflects the transport capacity of the barriers, especially the ratio indicating the similarities and differences of the transport capacity between the pig BAB and BCSFB, while the transporter protein amounts in the leptomeninges in the spinal region are not involved in the results. One of the most significant findings has been the protein amount of OAT1 in the leptomeninges, which was 125 ± 3 pmol and comparable to GLUT1 (134 ± 8 pmol), the highest expression among them all (Table IV). The protein amount of OAT3 in the leptomeninges was 54.8 ± 1.8 pmol, which was approximately half of OAT1. The protein amounts of OAT1 and OAT3 in the leptomeninges were 8–9-fold greater than those of the choroid plexus (Table IV). It is also an interesting finding that the protein amounts of organic cation transporter 2 (OCT2/*SLC22A2*) and MATE1 in the leptomeninges were 40.6 ± 1.2 pmol and 15.2 ± 0.9 pmol, respectively, and were 90-fold and 33-fold higher than those of choroid plexus (Table IV). The protein amounts of MDR1 and BCRP in the leptomeninges were 25.1 ± 0.9 pmol and 36.1 ± 0.9 pmol, respectively, which play significantly important roles as a functional barrier decreasing concentrations of the endogenous and exogenous substrates in the CSF in the subarachnoid space.

**Table IV:** Comparison of Total Transporter Protein Expression Between Cerebral Leptomeninges and Choroid Plexus in Pig

Transporter	Protein expression per head (pmol/pig cerebrum)		Ratio
	cerebral leptomeninges	choroid plexus	leptomeninges /choroid plexus
Drug efflux transporter			
MDR1/*ABCB1*	25.1 ± 0.9	13.6 ± 0.1	1.84
BCRP/*ABCG2*	36.1 ± 0.9	8.42 ± 0.20	4.29
Organic anion transporter			
OAT1/*SLC22A6*	125 ± 3	14.0 ± 0.2	8.94
OAT3/*SLC22A8*	54.8 ± 1.8	7.06 ± 0.35	7.76
MRP1/*ABCC1*	ULQ(<5.62)	8.04 ± 0.18	<0.699
MRP3/*ABCC3*	3.80 ± 0.12	4.56 ± 0.11	0.833
MRP4/*ABCC4*	4.07 ± 0.21	2.65 ± 0.08	1.54
OATP1A2/*SLCO1A2*	ULQ (<8.43)	40.1 ± 0.6	<0.21
OATP2B1/*SLCO2B1*	4.72 ± 0.16	2.99 ± 0.07	1.58
OATP3A1/*SLCO3A1*	ULQ (<1.63)	10.9 ± 0.1	<0.149
Organic cation transporter			
MATE1/*SLC47A1*	15.2 ± 0.9	0.464 ± 0.042	32.8
OCT2/*SLC22A2*	40.6 ± 1.2	0.452 ± 0.021	89.8
OCTN2/*SLC22A5*	ULQ (<2.65)	12.6 ± 0.2	<0.211
Energy source transporter			
GLUT1/*SLC2A1*	134 ± 8	112 ± 1	1.20
MCT1/*SLC16A1*	15.8 ± 0.9	13.1 ± 0.3	1.21
Thyroid hormone transporter			
MCT8/*SLC16A2*	10.0 ± 0.6	8.01 ± 0.10	1.25
Amino acid transporter			
xCT/*SLC7A11*	72.8 ± 3.3	11.2 ± 0.3	6.50
ATA2/*SLC38A2*	9.40 ± 0.69	ULQ (<5.49)	>1.71
Peptide transporter			
PEPT2/*SLC15A2*	16.4 ± 0.5	2.83 ± 0.09	5.79

Values were obtained from Table II in the reference (36)

*ULQ*, under limit of quantification; *ATA*, amino acid transporter

Considering the physiological role of the transporters in the barrier, it is crucial to clarify the polarized localization of the transporter proteins in the plasma membrane, i.e., either the CSF side or the blood side, or both sides (Fig. 2C, D). We have established a method to solve the subject by quantifying transporter proteins including marker proteins located either the CSF side and blood side for the plasma membrane fractions separately isolated for the CSF side rich and the blood side rich by the density gradient separation method (8, 36). As the specific peptide of each transporter protein is quantified by the LC-MS/MS, the possibilities of identifying any transporter proteins, which may belong to the same transporter family, can be excluded. The other advantage is the multiplex analysis, i.e., several transporter proteins can be clarified simultaneously (8). We have applied the method for analysis of leptomeninges in the pig. OAT1, OAT3, OCT2, and peptide transporter 2 (PEPT2/*SLC15A2*) were identified in the plasma membrane of CSF facing side in the leptomeninges (Fig. 2D), suggesting that those substrates, organic anions, organic cations, and di- or tri-peptides would be transported at the plasma membrane of the leptomeninges. Interestingly, MDR1, BCRP, MATE1, multidrug resistance-associated protein 4 (MRP4/*ABCC4*), and OATP2B1/*SLCO2B1* were identified in the plasma membrane of blood facing side in the leptomeninges (Fig. 2D), suggesting that substates of MDR1, BCRP, MRP4, and OATP2B1 are pumped out from the arachnoid epithelial cells to the circulating blood, which will cause significant decrease of the substrate concentration in the CSF.

Considering the abundant expression of these transporter proteins in the leptomeninges listed in Table IV, these transporters play important roles of the BAB as a dynamic interface for the exchange of nutrients and xenobiotics between the circulating blood and the CSF in the subarachnoid space. As OCT2 and MATE1 could transport substrates bi-directionally at the plasma membrane, there would be possibilities that substrates of these transporters are carried from the circulating blood to the CSF in the subarachnoid space (Figs. 1 and 2D). Further studies would be necessary to clarify the transport direction of these substrates including OATP2B1 across the BAB, which would be important for the safety assessment to the CNS including transporter-mediated drug-drug interaction. The transporter protein amounts of the BAB were significantly different from those of the BCSFB (Table IV), demonstrating significantly different physiological roles of two barriers for the nutrients and xenobiotics turnover in the CSF.

## Structural Design of Transporter-Utilizing Compounds

### Understanding of Transport Function Is Critical for the Rational Design of (Pro)Drugs to Be Efficiently Delivered to the CNS

As described above, there are several SLCs and ABCs not only at the BBB (Fig. 2A), but also at the BSCB, BCSFB, and BAB, (Fig. 2B, C, D, respectively) participating in the transporter of endogenous compounds and metabolites in and out of the brain. However, there are several carriers also transporting particular drugs, either into the brain (influx) or out of the brain (efflux) and thus, determining the drug exposure in the brain (Table V). A very well-known example of SLC function at the BBB is LAT1/*SLC7A5* carrying the anti-parkinsonian drug L-dopa and the anti-epileptic drug gabapentin (Table V)(79). Another important family of drug carriers at the BBB are organic anion transporting polypeptides (OATPs); although there are only very limited data available demonstrating their clinically relevant role in brain drug disposition to date, they are known to be responsible for carrying several drugs, including statins, dopamine receptor antagonists and opioid conjugates across the BBB, mainly via OATP2B1/*SLCO2B1* (39–41) and OATP1A2/*SLCO2A1* (39, 41–43) (Table V) (Fig. 2A). Notably, OATP-family is also expressed widely in other peripheral organs, e.g., OATP1B1/*SLCO1B1* and OAPT1B3/*SLCO1B3* are highly expressed in the liver (80, 81). Moreover, the substrate specificities of OATPs overlap, and thus, targeting CNS-acting drugs to their site of action via OATPs and simultaneously avoiding the peripheral exposure, can be very challenging. Furthermore, the overlapping substrate specificities with efflux transporters at the CNS barriers are needed to be taken into consideration at the early stages of drug design to gain maximal brain drug delivery of novel CNS-acting drugs (Table VI).

**Table V:** Most Important Membrane Transporters for Brain Drug Delivery. The Data Have Been Collected from Uniprot Proteome Databases and the Database Mentioned in the Reference (82), Unless Otherwise Stated

		Gene name(s)	Endo-genous substrate(s)	Main Physiological role	Drug substrates / inhibitors(^#^)	Expression at the Brain barriers			
						BBB	BSCB	BCSFB	BAB
Efflux transporters	ABCs	*ABCA2*	Lipid (cholesterol)	Sphingolipid homeostasis - Cholesterol homeostasis – Transport across the BBB	–	+ (a)			
		*ABCA8*	Tauro-cholate and estrone sulphate	Sphingomyelin production – Lipid transport - Cholesterol homeostasis	–	+ (a)	+ (b)	+ (c)	
		*ABCB1* (MDR1)	Phospho-lipids	Xenobiotic transport across blood-brain barrier - Phospholipid translocation	Verapamil, Saquinavir, Reserpine, Nifedipine, Mifepristone, Dexamethasone, Digoxin, Trimethoprim, Progesterone, Tacrolimus, Phenobarbital, Tamoxifen, Asciminib, Octreotide, Temozolomide, Lamotrigine	+ (a)	+ (b)	+ (c)	+p (d)
		*ABCC1* (MRP1)	GSH, leukotriene C4, estradiol glucuronide	GSH transport - Leukotriene metabolic process - Cobalamin transport	Grepafloxacin, Saquinavir, Dactinomycin, Zoledronic acid, Atorvastatin, Saxagliptin, Acemetacin, Prasterone			+ (c)	
		*ABCC2 (MRP2)*	glucuronideand GSH conjugates	transport across blood-brain barrier – transport of thyroid hormone, leukotriene and bil acids	Vincristine, Methotrexate, Saquinavir, Ritonavir and Indinavir	+ (e)			
		*ABCC3* (MRP3)	Bilirubin di-glucuronide, Estradiol glucuronide, GSH conjugates	Canalicular bile acid transport – Leukotriene transport	Gadoxetic acid, Ezetimibe, Raloxifene, Fexofenadine, Methotrexate, Etoposide, Lamivudine, Fluorouracil				+p (d)
		*ABCC4* (MRP4)	cAMP, cGMP, Bile acids, Steroids, GSH, Prosta-glandins	Xenobiotic transport across blood-brain barrier - Cellular communication and signalling – Prostaglandin and GSH transport	GSH, Dinoprostone, Atorvastatin, Oseltamivir, Alprostadil, Cefazolin, Nateglinide, Fluorouracil, Raloxifene, Prasterone	+ (a)		+ (c)	
		*ABCC8*		Regulation of insulin secretion - Potassium ion transport		+ (a)			
		*ABCG2* (BCRP)	Wide variety of physiological compounds e.g., protopor-phyrin IX, sphingosine-1-P	Xenobiotic transport across blood-brain barrier – Biotin transport – Cellular detoxification – Heme biosynthetic process	Topotecan, Glyburide, Pravastatin, Doxorubicin, Mitoxantrone, Prazosin, Etoposide, Cerivastatin, Tamoxifen, Sumatriptan, Alvocidib, Ivermectin, Oxaliplatin, Leflunomide, Mycophenolate mofetil	+ (a)	+ (b)	+ (c)	+p (d)
Organic anion transporting polypeptide**s**	OATPs	*SLCO* *1A2* (OATP 1A2)	Conjugated and unconjugated bile acids	Recycling of bile acids and salts	Pravastatin, Enalapril, Rocuronium, Budesonide, Levofloxacin, Indomethacin, Deoxycholic acid, Fexofenadine	+			
		*SLCO* *2B1* (OATP 2B1)	Prosta-glandins (PGD2, PGE1, PGE2), leukotriene C4, thrombo-xane B2 and iloprost	Recycling of bile acids and salts - Heme catabolic process	Tolbutamide, Ibuprofen, Salicylic acid, Montelukast, Simeprevir Opicapone, Pravastatin, Dinoprostone, Fexofenadine	+			+p (d)
		*SLCO* *3A1* (OATP 3A1)	Estrone-3-sulfate, PGE1, PGE2, vasopressin, thyroxine	Transport across the BBB - Prostaglandin transport	Safinamide, Dinoprostone, Methotrexate, Iloprost, Conjugated estrogens, Alprostadil^#^			+ (c)	
		Other potential members such as *SLCO1A1, SLCO1A4, SLCO1C1, SLCO1B1, SLCO1B3, SLCO1B7, SLCO2A1, SLCO4A1, SLCO4C1, SLCO5A1 & SLCO6A1*							
Organic anion transporters	OATs	*SLC* *22A6* (OAT1)	Glutarate	Alpha-ketoglutarate transport - Renal tubular secretion	Didanosine, Famotidine, Probenecid, Lamivudine, Latanoprost, Furosemide				+p (d)
		*SLC* *22A8* (OAT3)	Estrone 3-sulfate	Excretion/ detoxification of endogenous and exogenous organic anions in brain and kidney	Valaciclovir, Oseltamivir, Saxagliptin, Allopurinol, Avibactam, Cefdinir, Edaravone, Sitagliptin, Budesonide, Ibuprofen^#^, Indomethacin^#^, Diclofenac^#^			+ (c)	+p (d)
Organic cation transport	OCTs	*SLC* *22A2* (OCT2)	Dopamine, nor-adrenaline, serotonin, choline	Transport across the BBB - Neurotransmitter clearance	Dalfampridine, Dofetilide, Terbutaline, Histamine, Amantadine, Metformin, Memantine, Pramipexole, Reserpine, Lamivudine, Amiloride^#^, Amiodarone^#^				+p (d)
Multidrug and toxin extrusion	MATEs	*SLC* *47A1* (MATE1)	Estrone sulfate	Secretion of cationic drugs - Transport of bile salts and organic acids	Cefradine, Metformin, Cimetidine, Cephalexin, Acyclovir, Ganciclovir Abemaciclib, Brigatinib, Relebactam, Fosdenopterin,, Famotidine^#^, Verapamil^#^			+ (c)	+p (d)
Amino acid transporters	EAATs	*SLC1A3* (EAAT1)	L-Glu, L-Asp & D-Asp	Glutamate synaptic transmission	Glu and Asp analogues	+ (a)	+ (b)	+ (c)	
		*SLC1A2* (EAAT2)				+ (b)	+ (b)		
	ASCTs	*SLC1A4* (ASCT1)	Ala, Ser, Cys and Thr	D-Ser synaptic transmission	–	+			
		*SLC1A5* (ASCT2)	Broad substrate specificity	Nutritional and developmental functions	Fluciclovine (^18^F), Serine conjugates	+ (a)			
	NAT	*SLC* *6A12* (BGT1)	Betaine and GABA	GABAergic transmission	Guvacine^#^	+ (a)			
	CATs	*SLC7A1* (CAT1)	Cationic AAs (Arg, Lys and Orn)	Transport of Arg - Signalling pathways such as mTORC and activation of macrophages	–	+ (a)		+ (c)	
	HATs	*SLC7A5* (LAT1)	Large neutral AAs.	Activation of mTOR signalling pathways – transport of thyroid hormones	Levodopa, Pregabalin, Baclofen, Gapepentin	+ (a)			
		*SLC7A11* (xCT)	Cys, Glu	Regulation of Cys and Glu metabolic processes	Sulfasalazine^#^, Thimerosal^#^				+p (d)
	SNATs	*SLC* *38A2* (SNAT2)	Small neutral AAs (L-Ala, L-Cys, L-Ser, L-His, L-Gln and L-Met)	Transport AAs across the BBB and placental barrier – Glutamate Neurotransmitter Cycle	–	+			+
		*SLC* *38A3* (SNAT3)	Gln, His, Asn, Ala	Regulation of Gln/Glu cycle - Nutritional and developmental functions	–	+ (b)	+ (b)		
	PEPT	*SLC* *15A2* (PEPT2)	Oligo-peptides & Dipeptides	Transport across BBB – Innate immune response	Quinapril, Ubenimex, Valaciclovir, Bestatin, Cefadroxil, Amoxicillin^#^, Chlorpropamide^#^				+p (d)
Monocarboxylate transporters	MCTs	*SLC* *16A1* (MCT1)	Lactate, pyruvate, branched chain oxo acids	Pyruvate metabolic process - glucose homeostasis – Transport across BBB	Salicylic acid, Foscarnet, Pravastatin, Probenecid^#^, Niflumic acid^#^	+ (a)	+ (b)	+ (c)	+p (d)
		*SLC* *16A3* (MCT4)			α-Ketoisovalerate, Oxamic Acid, Pyruvic acid			+ (c)	+
		*SLC* *16A4* (MCT5)			–			+ (c)	
		*SLC* *16A2* (MCT8)	Thyroid hormone transporter	Transport across the BBB - Thyroid hormone metabolic process	Leucine, Thyroid-Porcine, Levothyroxine^#^, Liotrix^#^	+ (b)	+ (b)	+ (c)	+p (d)
Glucose transporter	GLUTs	*SLC2A1* (GLUT1)	Glucose, aldoses	Most important energy carrier of the brain - Promotes retinal cone survival	Fludeoxyglucose (^18^F), Glucosamine, Resveratrol, Ascorbic acid, Butabarbital^#^, Etomidate^#^	+ (a)	+ (b)	+ (c)	+p (d)
Other influx transporters		*SLC5A6* (SMVT)	Panto-thenate, biotin and lipoate	Vitamin transporter activity – Transport across the BBB	Gabapentin enacarbil, Biotin	+ (b)	+ (b)		
		*SLC* *29A1* (ENT1)	Nucleosides (adenosine)	Nucleoside and neurotransmitter transport	Cytarabine, Fludarabine, Gemcitabine, Ribavirin, Fluorouracil, Cannabidiol^#^, Troglitazone^#^	+ (a)		+ (c)	
		*SLC* *29A4* (PMAT)	Monoamine neurotransmitt-ers	Neurotransmitter transport – Transport across the BBB	Metformin, Adenosine			+ (c)	
		*SLC* *44A1* (CTL1)	Choline	Membrane synthesis and myelin production – Transport across the BBB	Choline salicylate	+ (b)	+ (b)		
		*SLC* *44A2* (CTL2)				+ (b)	+ (b)		
		*SLC6A8* (CRT1)	Creatine	Creatine metabolic process	–	+		+ (c)	
		*SLC* *19A1* (RFC1)	Reduced folates	Folate transport – Transport across the BBB	Pralatrexate, Methotrexate, Levomefolic acid, Trimetrexate	+ (a)			
		*SLC* *27A1* (FATP1)	long-chain fatty acids (LCFA)	Transport of fatty acids – Cell signaling – Transport across the BBB	–	+			

*BBB*, Blood-brain barrier; *BSCB*, Blood spinal cord barrier; *BCSFB*, Blood-cerebrospinal fluid barrier; *BAB*, Blood-arachnoid Barrier; *AAs*, Amino acids; *GSH*, Glutathione; *L-Glu*, L-Glutamate; *L-Asp*, L-Aspartate; *D-Asp*, D-Aspartate; *Ala*, Alanine; *Ser*, Serine; *Cys*, Cysteine; *Thr*, Threonine; *Arg*, Arginine; *Lys*, Lysine; *Orn*, Ornithine; *L-His*, L-Histidine; *L-Gln*, L-Glutamine; *L-Met*, L-Methionine; *Asn*, Asparagine; *p*, pig

^#^refers to inhibitors; (a) data obtained from the reference (9); (b) data obtained from the reference (5); (c) data obtained from the reference (66); (d) data obtained from the reference (36); (e) data obtained from the reference (46)

**Table VI:** Challenges and Prospects in the Development of Transporter-Utilizing (Pro)Drugs

*In Vitro* • Exploration of expression and function of brain-selective enzymes to achieve site-selective bioconversion of prodrugs • Applying time-dependent experiments accompanied by computational methods to separate transported substrates from binding ligands • Evaluation of intracellular pharmacoproteomics to optimize the efficacy of transporter-utilizing compounds • Optimizing the affinity and the interactions of the substrates with adequate *in vitro* and computational methods (inducing dynamic process) to attain compounds that can compete with endogenous substrates for transporter utilization	
*In Vivo* • Utilization of quantitative proteomic data together with pharmacokinetic studies (pharmacoproteomics) to understand the drug disposition between the CNS and periphery • Characterization of transporter expression in the selected diseases during the early phase of the drug development phase to understand if there are changes in pharmacoproteomics as a part of the pathology • Discovering novel biomarkers related to transporter function to enable monitoring the disease conditions, progress, and effects of drug therapy • Exploring epigenetic regulation of the transcriptional and post-transcriptional mechanisms of drug transporters to predict the response of the CNS-therapies and attaining the personalized medicine • Studying and correlating the brain permeation data correctly from nocturnal rodents to diurnal humans to understand the effects of circadian rhythms at the CNS barriers	
*In Silico* • Understanding dynamic processes of protein by utilizing advanced computational methods, such as MDS, instead of using static protein models for protein-ligand interactions • Screening compounds towards several transporters and using machine learning for the prediction of overlapping substrate specificities and possible interactions with efflux transporter • Utilization of deep learning and generative methods in chemoinformatics and chemical biology in structural design and develop brain-targeted transporter-utilizing compounds with desired properties	

Thus, the drug development of novel CNS-acting drugs has been very challenging due to the improper drug delivery across the BBB resulting in a lack of efficacy and late-stage (Phase II-III clinical trials) failures (83, 84). Particularly, in drug discovery/development, a great challenge is to combine structural properties that are responsible for eliciting the pharmacological effects together with the acceptable pharmacokinetics and brain-targeting properties. With a prodrug approach, this can be overcome; with a temporal chemical modification, the prodrug can be targeted to the desired transporter at the CNS-barriers and be delivered more effectively into the brain. A well-known example of a transporter-utilizing brain-targeted prodrug is L-dopa that utilizes LAT1 for its BBB penetration (85) and is then bioconverted to dopamine by dopa decarboxylase (86). However, due to its premature bioconversion in the periphery, L-dopa needs to be given together with peripheral enzyme inhibitors, such as carbidopa (dopa decarboxylase inhibitor) and/or entacapone (catechol-*O*-methyltransferase (COMT)-inhibitor) (87, 88). Thus, the prodrugs should undergo biotransformation to their active parent drug forms, before they can interact with the final target proteins, although the biotransformation may take place before, during, or after BBB penetration, depending upon the bioactivation mechanism of the prodrug. Therefore, premature bioconversion rate and mechanism during the absorption and distribution need to be carefully evaluated to obtain a successful brain-targeted prodrug. Several enzymes, including hydrolyzing phosphatases (hydrolyzing e.g., anti-epileptic agent fosphenytoin and antiretroviral agent fosamprenavir), esterases (hydrolyzing e.g., neuraminidase inhibitor oseltamivir, anticoagulant dabigatran etexilate, acetylenic retinoid tazarotene, and antiglaucoma agent dipivefrin), and cytochrome P450 enzymes (responsible for oxidation of e.g., antineoplastic agents tegafur and nitrogen mustard cyclophosphamide) are known to participate in prodrug activation, but prodrugs can also be activated site-selectively via bacterial reductases (such as anti-inflammatory drug sulfasalazine) in the colon or chemically due to the change in hypoxic conditions of the tumor microenvironment (e.g., experimental anticancer agents tirapazamine and evofosfamide) (89, 90). One great advantage with prodrugs is the fact that they are suitable for several kinds of administration routes (89, 90), including intravenous infusion (fosphenytoin and tegafur) and particularly oral administration (fosamprenavir, oseltamivir, dabigatran etexilate, cyclophosphamide, and sulfasalazine), which is the preferred route for patients.

**Fig. 7:**
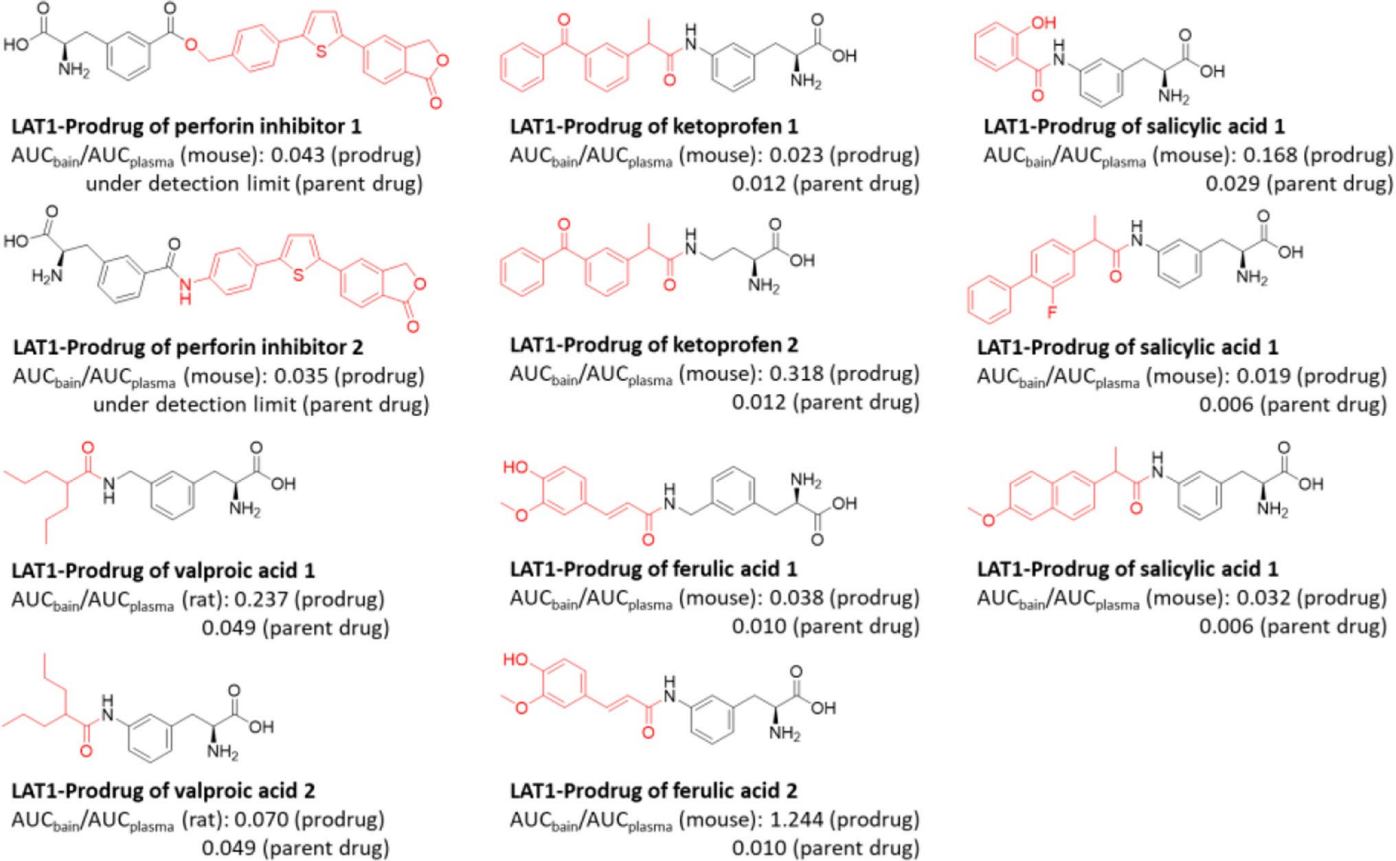
Structures of developed LAT1-utilizing prodrugs (parent drug highlighted with red color) and their brain-targeting effectiveness compared to their parent drugs reported as AUC_brain_/AUC_plasma_ values from the pharmacokinetic studies of either mice or rats.

Since CNS-diseases is one the greatest threats to public health and there has been a slowdown and withdrawal of pharmaceutical companies from CNS-drug development in past decades, there is an enormous social, clinical, and economic need for improved CNS-therapies (83, 84, 91). We have shown with several parent drugs, including anti-inflammatories (ketoprofen, flurbiprofen, naproxen, salicylic acid, anti-oxidants (ferulic acid), anti-epileptics (valproic acid), and investigational immunomodulators (perforin inhibitors), that an attachment of a cleavable amino acid promoiety to these parent drugs via a hydrolytic bond, and thus creating prodrugs, can significantly improve their brain uptake across the BBB via LAT1 (AUC_brain_/AUC_plasma_ ratio increased up to 124-times with prodrugs compared their parent drugs) (Fig. 7) (92–96). We have also systematically shown that LAT1-utilizing prodrugs can deliver the parent drugs into the brain parenchyma effectively, since neurons, astrocytes, and microglia express LAT1 protein (97, 98). Moreover, the LAT1-delivery was not changed at the BBB of mice having *APP/PSI* Alzheimer’s disease (AD) gene mutations of induced inflammation by lipopolysaccharide (LPS) or into the astrocytes isolated from the above-mentioned AD-transgenic mice or astrocytes induced with LPS *in vitro*, since the expression or function of LAT1 was not altered in these conditions (97, 99). The increased delivery (release) of the parent drugs into the brain parenchyma has been also shown to greatly improve the neuroprotective effects (100–103). However, it needs to be remembered the effective site-selective release of the parent drugs in brain parenchyma avoiding the systemic premature bioconversion should be carefully optimized, e.g., via prodrug bond selection, since there are more species-related variations in bioconverting enzyme functionalities than LAT1-mediated transport (104, 105). More importantly, there is a lack of knowledge of brain-selective prodrug bioconverting enzymes. Thus, if no brain-selective bioconversion is possible to be achieved, peripherally-acting (meaning not crossing BBB) enzyme inhibitors could be co-administered with the prodrug under the development. This is a highly important issue to be noted, particularly with carboxylesterases (CES), which are known to have higher expression levels in the rodent first-pass metabolism compared to humans (89, 103, 104). Therefore, the utilization of peripheral CES-inhibitors could provide more reliable IVIVE and translation to the human clinical situation when used together with prodrugs that are prematurely bioconverted in rodents.

Nevertheless, LAT1 is not selectively expressed at the BBB, instead, it has also been found in the spleen, testis, colon, kidney, liver, placenta, skeletal muscles (106–108), which also affects the pharmacokinetics and brain delivery of LAT1-utilizing prodrugs. Therefore, the expression profile of each transporter at the CNS compared to the peripheral tissues needs to be considered carefully, when developing brain-targeted transporter-utilizing compounds (Table VI). Moreover, LAT1 is not the only transporter to be utilized at the BBB, also GLUT1 and Na^+^-dependent vitamin C transporter (SVCT2/*SLC23A2*) have been successfully used by conjugating L-ascorbic acid to losartan or ibuprofen either directly or via a lipophilic thiamine disulfide system that can lock the compound into the brain after reduction of disulfide bond to thiazolium ion (109, 110). In the case of losartan, SVCT2/GLUT1-mediated increased brain exposure of the prodrug and released parent drug (losartan itself was not delivered into the brain at all) subsequently improved the locomotor activity and motor coordination in Parkinson’s Disease (PD) rat model. However, as we cannot exclude the peripheral exposure of these kinds of prodrugs in the other organs expressing SVCT2 and GLUT1, it would be highly important to evaluate both benefits of the CNS-effects and risks of the peripheral adverse reactions during the drug development process.

### Molecular Dynamics Simulations (MDS) Improve Understanding of the Dynamic Structure of Transporter and Substrate Structure Design

**Fig. 8:**
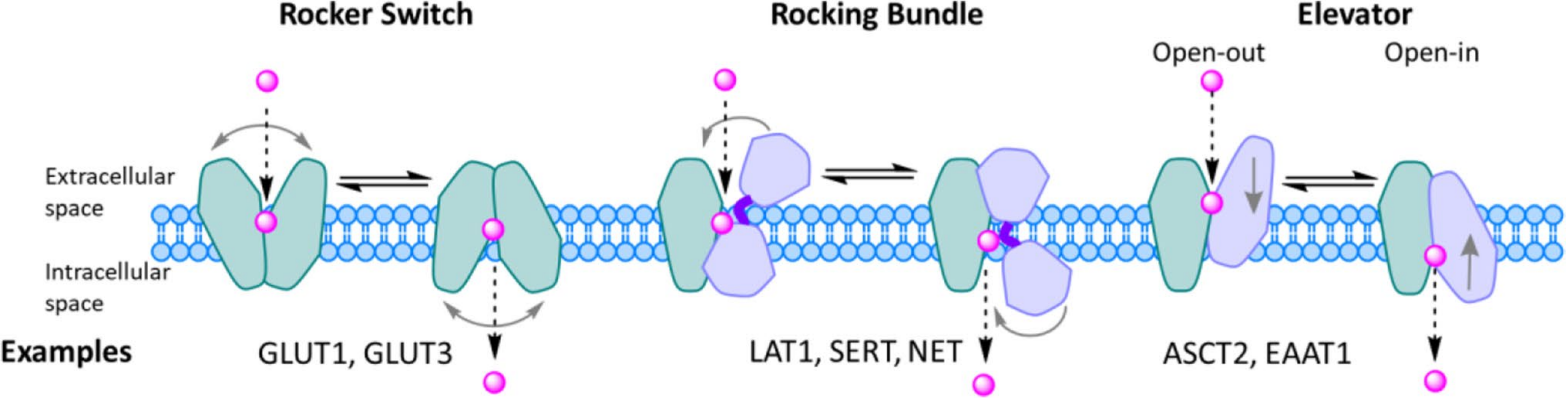
The alternating access transport mechanisms; 1) rocker switch (left), 2) rocking bundle (middle), and 3) elevator type (right), with their example transporters named below.

In very recent years, structural biology has rapidly and exponentially increased our understanding of membrane transporters (111–113). Recent advancements e.g., in X-ray crystallography and cryogenic electron microscopy (cryo-EM) have led to today’s high-resolution (< 2.5 Å) protein structures, which are rich in information. However, these structures are static and the transport process is a very dynamic process. Therefore, other techniques, such as computational methods, are needed to understand not only the binding of ligand to the target transporter, but also the translocation of substrates and their kinetic properties during the process (111). The dynamic process of transporters is generally described by the “alternating access” model (suggested by Oleg Jardetzky and Peter Mitchell already in the mid-1960s), in which the transporter alternates between outward and inward-facing conformations and has multiple intermediate states, like outward- and inward-occluded states (114, 115). To date, the improved computer power has enabled more detailed molecular dynamics simulations (MDS) and free energy calculations, and together with experimental transport data have helped us to understand and separate different kinds of mechanisms of this so-called “moving barrier” (116–119). These mechanisms for SLCs include (so far) 1) a rocker switch, 2) a rocking-bundle (or gated pore), and 3) an elevator-type mechanism (Fig. 8) (120–122).

**Fig. 9:**
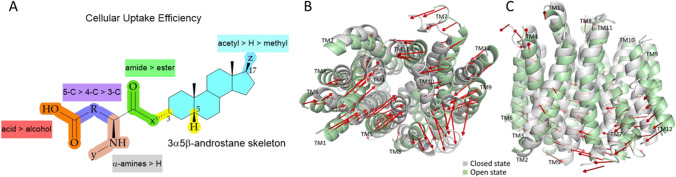
Structure-activity (function) relationships (SAR) of neurosteroids in relation to OATP1A2-mediated cellular uptake (**A**) and principal component analysis of OATP1A2-ligand complexes revealing two states open and closed at the intracellular view (**B**) and lateral view (**C**). The figures are modified versions of the ones described in the reference (20).

In the rocker switch mechanism that is a typical mechanism for glucose transporters (e.g., GLUT1 and GLUT3/*SLC2A3*), there are four distinct states; firstly the ligand binds to the transporter in its outward-open state, which causes the outer gate to close to form the second outward-occluded state followed by a rocker-switch movement to the third, inward-occluded state, and then, the ligand is finally released as the inner gate opens (Fig. 8; left) (114, 115, 120). This mechanism resembles roughly a V-shape architecture, while the second mechanism, the rocking-bundle resembles more like a K-shape architecture. In the rocking-bundle mechanism that is common for transporters such as LAT1 as well as serotonin and norepinephrine transporters SERT/*SLC6A4* and NET/*SLC6A2*, there are two main stages; the ligand binds roughly to the center on the interface between two domains in the outward-open state, which is closed by a thin gate, such as a salt bridge on the extracellular site and a thick gate on the intracellular site. Then, the scaffold domain (light green color in Fig. 8 middle) remains static, while the bundle domain (light purple color in Fig. 8 middle) goes through conformational changes, resulting in the inward-open state and the release of the ligand (114, 115, 121). In the elevator type mechanism that e.g., ASCT2/*SLC1A5* and EAAT1/*SLC1A3* uses, the transporter domain (light purple color in Fig. 8 right**)**, to which the ligand binds, is moved from outward-open state vertically (piston-like movement) within the membrane to form the inward-open state, while the oligomerization domain (light green color in Fig. 8 right) remains static (114, 115, 122). Thus, keeping in mind that a transporter can have different conformational states, it is easy to understand that docking compounds e.g. to the inward-open state may not result in the best substrate design and successful substrates (Table VI). Notably, this has been the case with LAT1 in the past, since the first cryo-EM structure was achieved from the inward-open state (123, 124). Thus, due to the current limitations of structure-based molecular modeling, and improvements in the data accuracy and treatment consistency, ligand-based molecular modeling approaches, such as 3-dimensional quantitative structure-activity relationship (3D-QSAR) and pharmacophore modeling, have retained their favor in predicting the interactions with the transporters based on their molecular properties (125, 126). We have recently found some critical structure-activity relationships (SAR) between novel neurosteroids and OATP1A2-mediated transport with the aid of the MDS (20). Structurally, a 3α5β-androstane core can be functionalized at both ends of C-3 and C-17 positions (Fig. 9A). In more detail, the amidic structures at the C-3 position next to the α-amine are favored over the corresponding esters, a terminal carboxylic acid is a definite requirement (over alcohol) and the length of the C-3 residue should be at least 5 carbons long to achieve efficient OATP1A2-mediated cellular uptake avoiding P-gp/BCRP/MRP-mediated effluxing out of the cells. The further principal component analysis of OATP1A2-ligand-bound complexes revealed two sets of extreme motions suggesting the preferred protein conformations for ligands (Fig. 9B, C). The most favorable structures displayed an open conformation toward the intracellular site and stabilized residues, namely Arg168, Glu172 (TM4), Glu200 (TM5), and Arg556 (TM11) on the transmembrane helices. Therefore, these influences of the compound structural features on the helical movements and thus, in opening-closing transition can be taken into account in the future design of OATP1A2-utilizing compounds. However, it was also noticed that the studied compounds may have two distinct binding sites in the same OATP (different transport efficiencies) or affinity towards different OATP subtypes, which was not differentiated in the study. Therefore, this together with the functionalization of the acetyl group at the C-17 position should be explored more thoroughly in the future.

### Appropriate Transport Kinetics Is Important for a Better Understanding of Transport Function

**Fig. 10:**
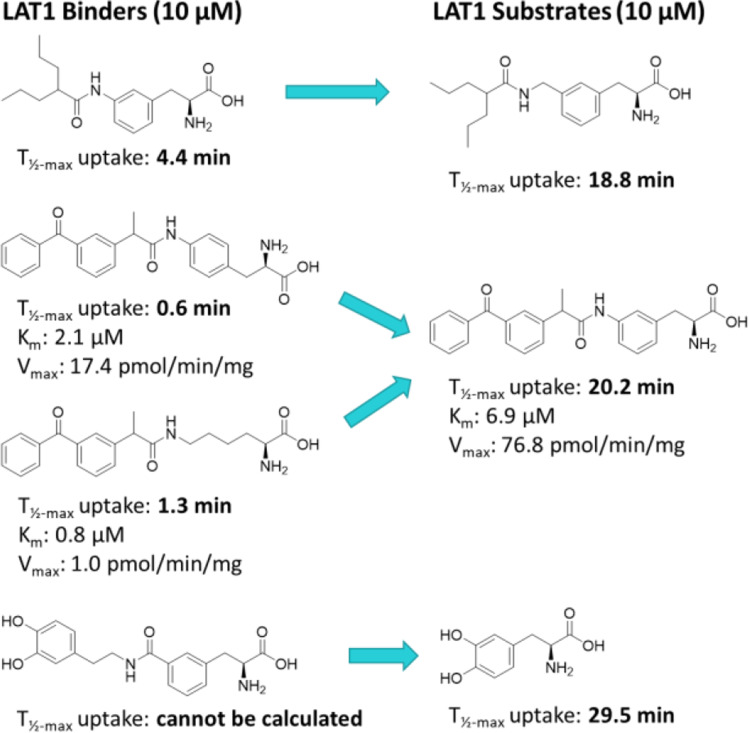
Molecular structures of LAT1-binders differentiated from LAT1-substrates according to their half-maximal uptake time (T_½_) studied at 10 μM concentration with LAT1-overexpressing MCF-7 human breast cancer cells. Additionally, ketoprofen compounds (in the middle) have also been provided with the Michaelis-Menten kinetic values (K_m_ and V_max_).

When choosing the target transporter to be utilized (Fig. 2) e.g., for brain drug delivery, biophysical and biochemical roles of the transporters are also needed to be considered carefully. Due to the similar features in the function, enzyme kinetics has been widely applied for the transporters, which may not be the most optimal approach (Table VI). Michaelis-Menten kinetics achieved by analyzing concentration-dependent uptake of substrates estimates the transporter capacity (V_max_; e.g., nmol/(min x mg protein)) and ligand’s affinity (to reach the half of the maximum V_max_) for the transporter (K_m_; μM) can be misleading, and transporters’ function should be characterized in a way that takes into account the translocation speed of the substrate across the membrane via the target transporter. Therefore, we have suggested that time-dependent uptake should be evaluated more closely, not only to find the most suitable time point for the concentration-dependent uptake assay, but also to understand how tightly the compounds are interacting with the transporter over time and if they can induce the alternating access mechanism that will enable the release of the compound from the transporter, e.g., in the cytosolic side (19). Thus, this kind of modified assay can help to differentiate the substrates (transported through the membrane) from binders (only binding to the transporter on the membrane), which is highly important to understand in order to achieve the highest possible drug response in the brain. We have, for example, found by that way that small amino acid-mimicking ligands may benefit from the elongation of the aliphatic side and expansion of the flexibility to achieve the required rocking-bundle mechanism of LAT1 (T_½-max_ increasing from 4 to 19 min) (Fig. 10) (19). In addition, attachment of the side chain to the *para*-position of the L-Phe and using aliphatic amino acid residues, such as L-Lys may support stronger binding and thus, inhibitory properties instead of being transported as a substrate via LAT1 (T_½-max_ 0.6–1.3 min *vs.* 20 min) (Fig. 10). Moreover, it was noticed that increasing the polarity of the side chain, particularly with larger compounds may increase the solvation effect at the outward-open state of the cavity and thus, not induce the required rocking-bundle mechanism of LAT1 (Fig. 10).

It should be noted also that any given compound is most likely a substrate not only a single transporter but to several of them. Working with transfected cell lines may hide this information, but also working with different inhibitors and native cells can result in confusing outcomes. We have seen that inhibiting LAT1-mediated uptake can actually increase the cellular accumulation of compounds, when they have been driven to another lower affinity, but higher capacity transporter, whereas LAT1 is a higher affinity, but lower capacity transporter (127–129). We have also found out that LAT1-utilizing prodrugs can have lower affinity at least for OATPs, but most likely also to some other yet un-identified amino acid transporter(s). Therefore, this can have an impact on the pharmacokinetics and brain delivery properties of these prodrugs; they may distribute to other organs, but then again, concomitant utilization of OATP1A2 or 2B1 at the BBB (Fig. 2A) can increase their brain uptake. Considering *in vivo* drug transport across the BBB, more efforts should be paid on the secondary and tertiary mechanisms in the drug development of each compound and utilize e.g., machine learning for the prediction of these interactions already during the early design of the compounds (Table VI).

The interference of endogenous substrates to transporter-utilizing compounds’ delivery or vice versa should also be carefully considered. The concomitant utilization of the same transporters by exogenous compounds may interfere with the endogenous substrate balance. In the case of amino acid transport via LAT1 into the brain, the basal levels of essential amino acids in the brain parenchyma were proved to be on a high enough level, and therefore, occupation of LAT1 temporarily by a slowly-reversible amino acid mimicking LAT1-inhibitor did not affect significantly brain amino acid homeostasis (130). Hence, the affinities of the endogenous and exogenous compounds towards the target transporter need to be in balance with the transportation speed through the transporter cavity. If the substrate has a very high affinity (e.g., ketoprofen prodrugs with L-Lys (K_m_ 0.8 μM) and L-Tyr (2.1 μM) promoieties in Fig. 10) and thus, high binding interactions with the transporter, it may not induce the dynamic process of the transporter (V_max_ 1.0 and 17.4 pmol/(min x mg protein), respectively). Therefore, the optimization (such as attachment of the parent compound to the *meta*-position of the L-Phe promoiety in Fig. 10; K_m_ 6.9 μM, V_max_ 76.8 pmol/(min x mg protein)), are crucial to be executed in the early phase of drug development of transporter-utilizing compounds (Table VI) (19, 127).

## Prospects

### Considering Multiple Factors Will Improve the Understanding of CNS Barrier Functions and Regulations, and Be Helpful for the Drug Delivery to the Brain

Notably, little is known about the life cycles of transporters and their intracellular functions before they are transferred to the plasma membrane or vice versa. For example, LAT1 is recruited by LAPRM4b to lysosomes, leading to the lysosomal accumulation of L-Leu and subsequent mTORC1 (mammalian target of rapamycin complex 1) activation (131). Therefore, LAT1-utilizing compounds can also be accumulated into lysosomes (129), which may affect the pharmacological response of the drugs if they are trapped into the lysosomes when their final targets are in the cytosolic site or they are needed to be transported across the epithelial cells. Moreover, there can also be species differences in intracellular transporter expression. For example, human ENT1 (hENT1) is today known to be responsible for mitochondrial toxicity of anti-hepatitis B agent, fialuridine (132). However, due to the lack of mitochondrial ENT1 expression in rodents (mice and rats) and thus, significant species differences between humans and mice, this was not predicted from the pre-clinical toxicity studies (133). Therefore, intracellular pharmacoproteomics of each drug candidate should be studied carefully during the preclinical phase (Table VI).

It has also been notified that some transporters can be down- or up-regulated as a part of disease pathogenesis (134, 135). For example, there are dozens of transporters already recognized, in which a single gene mutation is linked with diseases (Mendelian diseases), some of them affecting normal brain function. On the other hand, GLUT1 and LAT1 may be useful to deliver compounds into brain tumors, such as accumulating boron (^10^B) in the boron neutron capture therapy (BNCT), since GLUT1 and LAT1 are often overexpressed in many gliomas (136–138). The nuclear capture after low energy thermal neutron beam and subsequent fission reaction of ^10^B yields high-energy α-particles (^4^He) and ^7^Li that selectively destroy the cancer cells without affecting non-boron-containing healthy cells. Therefore, these expressional changes in the selected disease should be clarified in the very beginning when choosing the brain-delivering transporter to be utilized (Table VI).

To understand more of transporters’ role in the clinical context and their functional (or expression) changes in the disease, specific biomarkers should also be explored more broadly (Table VI) (139, 140). This may be challenging due to the overlapping substrate specificities of distinct transporters. In addition, several other criteria are needed to be met for the proposed endogenous compounds that could serve as valid biomarkers for transporters’ function, including sensitivity, robustness (measured from plasma or urine), and predictivity (141). Noteworthy, if reliable biomarkers are discovered, they can also be used to assess transporter-related DDIs. One way is the use of probe-drug cocktails, including several transporter substrates without mutual interaction, which are administered together with the drug candidate. This approach has been successfully used in the past with cytochrome P450-related DDIs and the field seems to be rapidly already emerging also in the case of the most common drug carriers (142–145). This gained knowledge should also be applied to the interactions at the BBB level. For example, although substrate specificities in the OATP-family overlap, levofloxacin could be used as an OATP1A2-selective substrate interacting at the BBB (146, 147). However, more mechanistic studies are required to establish selective OATP2B1-substrates (148), although naringin could serve as a probe substrate interacting with both OATP1A2 and 2B1 at the BBB (149, 150).

To predict the response of the CNS-therapy and attain personalized medicine, more focus should be paid to the inter-individual differences in the transporter expression at the CNS-barriers. There are already pieces of evidence that female rats have greater BBB permeability of taurocholate and atorvastatin, which are carried by Oatp1a4/*Slco1a4* (an ortholog for human OATP1A2), compared to the corresponding brain uptake of these compounds with male rats (151). A similar kind of trend has been seen in RNA expression of OATP1A2 collected from the human liver samples; females had approximately twice greater OATP1A2 mRNA expression compared to that in men (*P* < 0.05) (152). Therefore, more studies are warranted to elucidate the age-, race-, and gender-related effects on the pharmacoproteomics, not only OATP1A2 substrates but also other transporters at the CNS-barriers (Fig. 2, Tables V and VI). More importantly, epigenetic mechanisms and post-translational modifications (PTM) controlling the transporters’ life cycle are not well understood in many cases. For example, OATP1B1/*SLCO1B1* expression is known to be regulated via *N*-glycosylated and phosphorylated in the hepatocytes (153–156). However, it is not clear how these PTM affect the OATP1A2 or 2B1 expression at the CNS-barriers. Therefore, integrated analysis of global proteome, phosphoproteome, and glycoproteome could help in the interpretation of disease-related protein networks and facilitate personalized medicine (157).

Curiously, there is also emerging evidence of translational challenges with circadian rhythms at the CNS barriers (158, 159). It is already known that P-gp at the BBB is downregulated during the nighttime compared to the daytime, and thus making the brain drug permeation less restricted during the sleeping period (160–162). Unfortunately, the importance of circadian biology is rarely considered in pre-clinical studies, although the daytime executed experiments with nocturnal rodents can be very different from diurnal humans. Therefore, fundamental differences have been seen in neuroprotection in human patients with stroke compared to the used rodent models (159). However, it needs to keep in mind that other aspects of rodent models, such as age, hypertension, and metabolic disease needs to be matched with clinical populations. Thus, circadian rhythms that are affected by genetic and environmental factors, may not be the exclusive reason for translational failures, but they should be considered more carefully in future CNS-drug development (Table VI).

Finally, in transporter-utilizing (pro)drug design deep learning and generative methods in chemoinformatics and chemical biology should be utilized more effectively to create molecules for the desired biological properties and not vice versa (to find the properties for established molecules)(163) (Table VI). These methods require a deep understanding of chemical biology, computational sciences, and bioinformatics, in parallel. As the methods and the calculating power in the computational field are currently developing at an accelerated speed, it will allow increasingly larger data to be processed much faster than in the past. We also presume that the utilization of data mining in pharmacoproteomics will help in finding changes and correlations to predict the role of transporters in diseases and clinical outcomes. Together with the computer-aided drug design and development, it is believed that this will improve the success rate of future discovery and development of CNS-drugs.

## Conclusions

In conclusion, it is very important to include the drug delivery aspects early in the brain drug discovery and development process. By combining pharmacoproteomics of the CNS barriers together with computational drug design, we can influence the brain-targeting properties of compounds and finally achieve successful brain-targeted drug candidates for clinical use. When developing brain-targeted (pro)drugs that take advantage of transporter-mediated cargo, several key elements need to be considered; 1) the expression level of the target transporter at the CNS barriers, 2) prediction of K_p,uu,brain_ based on the pharmacokinetic model using the transporter protein expression level and intrinsic transport activity 3) localization (CNS *vs.* periphery) and species differences in both transporters and possible bioconverting enzymes, 4) structural requirements that enable the dynamic transport process of the compounds, 5) transporter selectivity, and 6) changes in transporter expression and/or function due to the different stages of distinct diseases and due to the individual variations.

## Supplementary Information

ESM 1 (PDF 491 kb)

## ABBREVIATIONS

ABC: ATP-binding cassette
ASCT: Alanine-serine-cystine transporter
AUC: Area under drug concentration
BAB: Blood-arachnoid barrier
BBB: Blood-brain barrier
BCRP: Breast cancer resistance protein
BCSFB: Blood-cerebrospinal fluid barrier
BSCB: Blood-spinal cord barrier
CES: Carboxylesterases
CNS: Central nervous system
cryo-EM: Cryogenic electron microscopy
CSF: Cerebrospinal fluid
COMT: Catechol-*O*-methyltransferase
CTL: Choline transporter-like transporter
CNT: Concentrative nucleoside transporter
DDI: Drug-drug interaction
EAAT: Excitatory amino acid transporter
GLUT: Glucose transporter
IVIVE: *In vitro-in vivo* extrapolation
LAT1: L-type amino acid transporter 1
MATE: Multidrug and toxin extrusion
MCT: Monocarboxylic acid transporter
MDR: Multidrug resistance protein
MDS: Molecular dynamics simulation
MRP: Multidrug resistance-associated protein
NCE: New chemical entities
NET: Norepinephrine transporter
OAT: Organic anion transporter
OATP: Organic anion transporting polypeptide
OCT: Organic cation transporter
P-gp: P-glycoprotein
SERT: Serotonin transporter
SLC: Solute carrier
SMVT: Sodium-dependent multi-vitamin transporter
SNAT: Sodium-coupled neutral amino acid transporter
3D-QSAR: 3-dimensional quantitative structure-activity relationship
4F2hc: 4F2 heavy chain

## References

[1] Kola I, Landis J (2004). Can the pharmaceutical industry reduce attrition rates?. Nat Rev Drug Discov.

[2] Choi DW, Armitage R, Brady LS, Coetzee T, Fisher W, Hyman S, Pande A, Paul S, Potter W, Roin B, Sherer T (2014). Medicines for the mind: policy-based "pull" incentives for creating breakthrough CNS drugs. Neuron..

[3] de Lange EC, Hammarlund-Udenaes M (2015). Translational aspects of blood-brain barrier transport and central nervous system effects of drugs: from discovery to patients. Clin Pharmacol Ther.

[4] Kamiie J, Ohtsuki S, Iwase R, Ohmine K, Katsukura Y, Yanai K, Sekine Y, Uchida Y, Ito S, Terasaki T (2008). Quantitative atlas of membrane transporter proteins: development and application of a highly sensitive simultaneous LC/MS/MS method combined with novel in-silico peptide selection criteria. Pharm Res.

[5] Uchida Y, Yagi Y, Takao M, Tano M, Umetsu M, Hirano S, Usui T, Tachikawa M, Terasaki T (2020). Comparison of absolute protein abundances of transporters and receptors among blood-brain barriers at different cerebral regions and the blood-spinal cord barrier in humans and rats. Mol Pharm.

[6] Hoshi Y, Uchida Y, Tachikawa M, Inoue T, Ohtsuki S, Terasaki T (2013). Quantitative atlas of blood-brain barrier transporters, receptors, and tight junction proteins in rats and common marmoset. J Pharm Sci.

[7] Ito K, Uchida Y, Ohtsuki S, Aizawa S, Kawakami H, Katsukura Y, Kamiie J, Terasaki T (2011). Quantitative membrane protein expression at the blood-brain barrier of adult and younger cynomolgus monkeys. J Pharm Sci.

[8] Kubo Y, Ohtsuki S, Uchida Y, Terasaki T (2015). Quantitative determination of luminal and Abluminal membrane distributions of transporters in porcine brain capillaries by plasma membrane fractionation and quantitative targeted proteomics. J Pharm Sci.

[9] Uchida Y, Ohtsuki S, Katsukura Y, Ikeda C, Suzuki T, Kamiie J, Terasaki T (2011). Quantitative targeted absolute proteomics of human blood-brain barrier transporters and receptors. J Neurochem.

[10] Pan Y, Hsu V, Grimstein M, Zhang L, Arya V, Sinha V, Grillo JA, Zhao P (2016). The application of physiologically based pharmacokinetic modeling to predict the role of drug transporters: scientific and regulatory perspectives. J Clin Pharmacol.

[11] Grimstein M, Yang Y, Zhang X, Grillo J, Huang SM, Zineh I, Wang Y (2019). Physiologically based pharmacokinetic modeling in regulatory science: an update from the U.S. Food and Drug Administration's Office of Clinical Pharmacology. J Pharm Sci.

[12] Taskar KS, Pilla Reddy V, Burt H, Posada MM, Varma M, Zheng M, Ullah M, Emami Riedmaier A, Umehara KI, Snoeys J, Nakakariya M, Chu X, Beneton M, Chen Y, Huth F, Narayanan R, Mukherjee D, Dixit V, Sugiyama Y, Neuhoff S (2020). Physiologically-based pharmacokinetic models for evaluating membrane transporter mediated drug-drug interactions: current capabilities, case studies, future opportunities, and recommendations. Clin Pharmacol Ther.

[13] Uchida Y, Ohtsuki S, Kamiie J, Terasaki T (2011). Blood-brain barrier (BBB) Pharmacoproteomics: reconstruction of in vivo brain distribution of 11 P-glycoprotein substrates based on the BBB transporter protein concentration, in vitro intrinsic transport activity, and unbound fraction in plasma and brain in mice. J Pharmacol Exp Ther.

[14] Uchida Y, Ohtsuki S, Terasaki T (2014). Pharmacoproteomics-based reconstruction of in vivo P-glycoprotein function at blood-brain barrier and brain distribution of substrate verapamil in pentylenetetrazole-kindled epilepsy, spontaneous epilepsy, and phenytoin treatment models. Drug Metab Dispos.

[15] Uchida Y, Wakayama K, Ohtsuki S, Chiba M, Ohe T, Ishii Y, Terasaki T (2014). Blood-brain barrier pharmacoproteomics-based reconstruction of the in vivo brain distribution of P-glycoprotein substrates in cynomolgus monkeys. J Pharmacol Exp Ther.

[16] Kodan A, Futamata R, Kimura Y, Kioka N, Nakatsu T, Kato H, Ueda K (2021). ABCB1/MDR1/P-gp employs an ATP-dependent twist-and-squeeze mechanism to export hydrophobic drugs. FEBS Lett.

[17] Türková A, Zdrazil B (2019). Current advances in studying clinically relevant transporters of the solute carrier (SLC) family by connecting computational modeling and data science. Comput Struct Biotechnol J.

[18] Jaramillo-Martinez V, Urbatsch IL, Ganapathy V (2021). Functional distinction between human and mouse sodium-coupled citrate transporters and its biologic significance: an attempt for structural basis using a homology modeling approach. Chem Rev.

[19] Kärkkäinen J, Laitinen T, Markowicz-Piasecka M, Montaser A, Lehtonen M, Rautio J, Gynther M, Poso A, Huttunen KM (2021). Molecular characteristics supporting l-type amino acid transporter 1 (LAT1)-mediated translocation. Bioorg Chem.

[20] Adla SK, Tonduru AK, Kronenberger T, Kudova E, Poso A, Huttunen KM. Neurosteroids: Structure-uptake relationships and computational modeling of organic anion transporting polypeptides (OATP)1A2. Molecules. 2021;26(18). 10.3390/molecules26185662 PMC8472597 34577133

[21] Kaczor AA, Karczmarzyk Z, Fruziński A, Pihlaja K, Sinkkonen J, Wiinämaki K, Kronbach C, Unverferth K, Poso A, Matosiuk D (2014). Structural studies, homology modeling and molecular docking of novel non-competitive antagonists of GluK1/GluK2 receptors. Bioorg Med Chem.

[22] Lüders E, Steinmetz H, Jäncke L (2002). Brain size and grey matter volume in the healthy human brain. Neuroreport..

[23] Sahar A (1972). Choroidal origin of cerebrospinal fluid. Isr J Med Sci.

[24] Thorne RG (2014). Primer on central nervous ststem structure/function and the vasculature, ventricular system, and fluids of the brain.

[25] Johanson C, Stopa E, McMillan P, Roth D, Funk J, Krinke G (2011). The distributional nexus of choroid plexus to cerebrospinal fluid, ependyma and brain: toxicologic/pathologic phenomena, periventricular destabilization, and lesion spread. Toxicol Pathol.

[26] Gupta A, Chatelain P, Massingham R, Jonsson EN, Hammarlund-Udenaes M (2006). Brain distribution of cetirizine enantiomers: comparison of three different tissue-to-plasma partition coefficients: K(p), K(p,u), and K(p,uu). Drug Metab Dispos.

[27] Adachi Y, Suzuki H, Sugiyama Y (2001). Comparative studies on in vitro methods for evaluating in vivo function of MDR1 P-glycoprotein. Pharm Res.

[28] Jonker JW, Buitelaar M, Wagenaar E, Van Der Valk MA, Scheffer GL, Scheper RJ, Plosch T, Kuipers F, Elferink RP, Rosing H, Beijnen JH, Schinkel AH (2002). The breast cancer resistance protein protects against a major chlorophyll-derived dietary phototoxin and protoporphyria. Proc Natl Acad Sci U S A.

[29] Doyle LA, Yang W, Abruzzo LV, Krogmann T, Gao Y, Rishi AK, Ross DD (1998). A multidrug resistance transporter from human MCF-7 breast cancer cells. Proc Natl Acad Sci U S A.

[30] Braun C, Sakamoto A, Fuchs H, Ishiguro N, Suzuki S, Cui Y, Klinder K, Watanabe M, Terasaki T, Sauer A (2017). Quantification of transporter and receptor proteins in dog brain capillaries and choroid plexus: relevance for the distribution in brain and CSF of selected BCRP and P-gp substrates. Mol Pharm.

[31] Tachikawa M, Watanabe M, Hori S, Fukaya M, Ohtsuki S, Asashima T, Terasaki T (2005). Distinct spatio-temporal expression of ABCA and ABCG transporters in the developing and adult mouse brain. J Neurochem.

[32] Kalvass JC, Maurer TS (2002). Influence of nonspecific brain and plasma binding on CNS exposure: implications for rational drug discovery. Biopharm Drug Dispos.

[33] Maurer TS, Debartolo DB, Tess DA, Scott DO (2005). Relationship between exposure and nonspecific binding of thirty-three central nervous system drugs in mice. Drug Metab Dispos.

[34] Liu X, Smith BJ, Chen C, Callegari E, Becker SL, Chen X, Cianfrogna J, Doran AC, Doran SD, Gibbs JP, Hosea N, Liu J, Nelson FR, Szewc MA, Van Deusen J (2006). Evaluation of cerebrospinal fluid concentration and plasma free concentration as a surrogate measurement for brain free concentration. Drug Metab Dispos.

[35] Yaguchi Y, Tachikawa M, Zhang Z, Terasaki T (2019). Organic anion-transporting polypeptide 1a4 (Oatp1a4/Slco1a4) at the blood-arachnoid barrier is the major pathway of Sulforhodamine-101 clearance from cerebrospinal fluid of rats. Mol Pharm.

[36] Uchida Y, Goto R, Takeuchi H, Luczak M, Usui T, Tachikawa M, Terasaki T (2020). Abundant expression of OCT2, MATE1, OAT1, OAT3, PEPT2, BCRP, MDR1, and xCT transporters in blood-arachnoid barrier of pig and polarized localizations at CSF- and blood-facing plasma membranes. Drug Metab Dispos.

[37] Rao VV, Dahlheimer JL, Bardgett ME, Snyder AZ, Finch RA, Sartorelli AC, Piwnica-Worms D (1999). Choroid plexus epithelial expression of MDR1 P glycoprotein and multidrug resistance-associated protein contribute to the blood-cerebrospinal-fluid drug-permeability barrier. Proc Natl Acad Sci U S A.

[38] Yasuda K, Cline C, Vogel P, Onciu M, Fatima S, Sorrentino BP, Thirumaran RK, Ekins S, Urade Y, Fujimori K, Schuetz EG (2013). Drug transporters on arachnoid barrier cells contribute to the blood-cerebrospinal fluid barrier. Drug Metab Dispos.

[39] Bronger H, Konig J, Kopplow K, Steiner HH, Ahmadi R, Herold-Mende C, Keppler D, Nies AT (2005). ABCC drug efflux pumps and organic anion uptake transporters in human gliomas and the blood-tumor barrier. Cancer Res.

[40] Schäfer AM, Zu M, Schwabedissen HE, Bien-Möller S, Hubeny A, Vogelgesang S, Oswald S, Grube M (2020). OATP1A2 and OATP2B1 are interacting with dopamine-receptor agonists and antagonists. Mol Pharm.

[41] Gao B, Vavricka SR, Meier PJ, Stieger B (2015). Differential cellular expression of organic anion transporting peptides OATP1A2 and OATP2B1 in the human retina and brain: implications for carrier-mediated transport of neuropeptides and neurosteriods in the CNS. Pflugers Arch.

[42] Gao B, Hagenbuch B, Kullak-Ublick GA, Benke D, Aguzzi A, Meier PJ (2000). Organic anion-transporting polypeptides mediate transport of opioid peptides across blood-brain barrier. J Pharmacol Exp Ther.

[43] Lee W, Glaeser H, Smith LH, Roberts RL, Moeckel GW, Gervasini G, Leake BF, Kim RB (2005). Polymorphisms in human organic anion-transporting polypeptide 1A2 (OATP1A2): implications for altered drug disposition and central nervous system drug entry. J Biol Chem.

[44] Uchida Y, Ito K, Ohtsuki S, Kubo Y, Suzuki T, Terasaki T (2015). Major involvement of Na(+) -dependent multivitamin transporter (SLC5A6/SMVT) in uptake of biotin and pantothenic acid by human brain capillary endothelial cells. J Neurochem.

[45] Tachikawa M, Uchida Y, Ohtsuki S, Terasaki T, Thorne R, de Lange L, Hammarlund-Udenaes M (2014). Recent progress inblood-brain barrier and blood-CSF barrier transport research: Pharmaceuticalrelevance for drug delivery to the brain. Drug delivery to the brain – physiological concepts.

[46] Luna-Munguia H, Salvamoser JD, Pascher B, Pieper T, Getzinger T, Kudernatsch M, Kluger G, Potschka H (2015). Glutamate-mediated upregulation of the multidrug resistance protein 2 in porcine and human brain capillaries. J Pharmacol Exp Ther.

[47] Bauer B, Hartz AM, Lucking JR, Yang X, Pollack GM, Miller DS (2008). Coordinated nuclear receptor regulation of the efflux transporter, Mrp2, and the phase-II metabolizing enzyme, GSTpi, at the blood-brain barrier. J Cereb Blood Flow Metab.

[48] Zhang Z, Tachikawa M, Uchida Y, Terasaki T (2018). Drug clearance from cerebrospinal fluid mediated by organic anion transporters 1 (Slc22a6) and 3 (Slc22a8) at arachnoid membrane of rats. Mol Pharm.

[49] Spector R, Johanson CE (1989). The mammalian choroid plexus. Sci Am.

[50] Abbott NJ (2004). Evidence for bulk flow of brain interstitial fluid: significance for physiology and pathology. Neurochem Int.

[51] Szentistványi I, Patlak CS, Ellis RA, Cserr HF (1984). Drainage of interstitial fluid from different regions of rat brain. Am J Phys.

[52] Nedergaard M (2013). Neuroscience. Garbage truck of the brain. Science.

[53] Iliff JJ, Wang M, Liao Y, Plogg BA, Peng W, Gundersen GA, Benveniste H, Vates GE, Deane R, Goldman SA, Nagelhus EA, Nedergaard M. A paravascular pathway facilitates CSF flow through the brain parenchyma and the clearance of interstitial solutes, including amyloid β. Sci Transl Med 2012;4(147):147ra111. 10.1126/scitranslmed.3003748 PMC3551275 22896675

[54] Hannocks MJ, Pizzo ME, Huppert J, Deshpande T, Abbott NJ, Thorne RG, Sorokin L (2018). Molecular characterization of perivascular drainage pathways in the murine brain. J Cereb Blood Flow Metab.

[55] Abbott NJ, Pizzo ME, Preston JE, Janigro D, Thorne RG (2018). The role of brain barriers in fluid movement in the CNS: is there a 'glymphatic' system?. Acta Neuropathol.

[56] Buck AK, Bommer M, Stilgenbauer S, Juweid M, Glatting G, Schirrmeister H, Mattfeldt T, Tepsic D, Bunjes D, Mottaghy FM, Krause BJ, Neumaier B, Döhner H, Möller P, Reske SN (2006). Molecular imaging of proliferation in malignant lymphoma. Cancer Res.

[57] Kindred JH, Koo PJ, Rudroff T (2014). Glucose uptake of the spinal cord in patients with multiple sclerosis detected by ^18^F-fluorodeoxyglucose PET/CT after walking. Spinal Cord.

[58] Sasaki K, Tachikawa M, Uchida Y, Hirano S, Kadowaki F, Watanabe M, Ohtsuki S, Terasaki T (2018). ATP-binding cassette transporter A subfamily 8 is a sinusoidal efflux transporter for cholesterol and taurocholate in mouse and human liver. Mol Pharm.

[59] Schnieder TP, Zhou Qin ID, Trencevska-Ivanovska I, Rosoklija G, Stankov A, Pavlovski G, Mann JJ, Dwork AJ (2019). Blood vessels and perivascular phagocytes of prefrontal white and gray matter in suicide. J Neuropathol Exp Neurol.

[60] Kumar G, Smith QR, Hokari M, Parepally J, Duncan MW (2007). Brain uptake, pharmacokinetics, and tissue distribution in the rat of neurotoxic N-butylbenzenesulfonamide. Toxicol Sci.

[61] Schlageter KE, Molnar P, Lapin GD, Groothuis DR (1999). Microvessel organization and structure in experimental brain tumors: microvessel populations with distinctive structural and functional properties. Microvasc Res.

[62] Ohtsuki S, Ikeda C, Uchida Y, Sakamoto Y, Miller F, Glacial F, Decleves X, Scherrmann JM, Couraud PO, Kubo Y, Tachikawa M, Terasaki T (2013). Quantitative targeted absolute proteomic analysis of transporters, receptors and junction proteins for validation of human cerebral microvascular endothelial cell line hCMEC/D3 as a human blood-brain barrier model. Mol Pharm.

[63] Uchida Y, Tachikawa M, Ohtsuki S, Terasaki T, Hammarlund-Udenaes M, de Lange E, Thorne R (2014). Blood–Brain Barrier (BBB) Pharmacoproteomics: A New Research Field Opened Up by Quantitative Targeted Absolute Proteomics (QTAP). Drug delivery to the brain, AAPS advances in the pharmaceutical sciences series.

[64] Wager TT, Villalobos A, Verhoest PR, Hou X, Shaffer CL (2011). Strategies to optimize the brain availability of central nervous system drug candidates. Expert Opin Drug Discov.

[65] Kodaira H, Kusuhara H, Fuse E, Ushiki J, Sugiyama Y (2014). Quantitative investigation of the brain-to-cerebrospinal fluid unbound drug concentration ratio under steady-state conditions in rats using a pharmacokinetic model and scaling factors for active efflux transporters. Drug Metab Dispos.

[66] Uchida Y, Zhang Z, Tachikawa M, Terasaki T (2015). Quantitative targeted absolute proteomics of rat blood-cerebrospinal fluid barrier transporters: comparison with a human specimen. J Neurochem.

[67] Qiu A, Jansen M, Sakaris A, Min SH, Chattopadhyay S, Tsai E, Sandoval C, Zhao R, Akabas MH, Goldman ID (2006). Identification of an intestinal folate transporter and the molecular basis for hereditary folate malabsorption. Cell..

[68] Dixon KH, Lanpher BC, Chiu J, Kelley K, Cowan KH (1994). A novel cDNA restores reduced folate carrier activity and methotrexate sensitivity to transport deficient cells. J Biol Chem.

[69] Cwiklinska M, Czogala M, Kwiecinska K, Madetko-Talowska A, Szafarz M, Pawinska K, Wieczorek A, Klekawka T, Rej M, Stepien K, Halubiec P, Lazarczyk A, Miklusiak K, Bik-Multanowski M, Balwierz W, Skoczen S (2020). Polymorphisms of SLC19A1 80 G>A, MTHFR 677 C>T, and tandem TS repeats influence pharmacokinetics, acute liver toxicity, and vomiting in children with acute lymphoblastic leukemia treated with high doses of methotrexate. Front Pediatr.

[70] Kotnik BF, Jazbec J, Grabar PB, Rodriguez-Antona C, Dolzan V (2017). Association between SLC19A1 gene polymorphism and high dose methotrexate toxicity in childhood acute lymphoblastic Leukaemia and non Hodgkin malignant lymphoma: introducing a haplotype based approach. Radiol Oncol.

[71] Shen J, Carcaboso AM, Hubbard KE, Tagen M, Wynn HG, Panetta JC, Waters CM, Elmeliegy MA, Stewart CF (2009). Compartment-specific roles of ATP-binding cassette transporters define differential topotecan distribution in brain parenchyma and cerebrospinal fluid. Cancer Res.

[72] Mason BL, Pariante CM, Thomas SA (2012). Changes in the brain accumulation of glucocorticoids in abcb1a-deficient CF-1 mice. J Neuroendocrinol.

[73] Kim WY, Benet LZ (2004). P-glycoprotein (P-gp/MDR1)-mediated efflux of sex-steroid hormones and modulation of P-gp expression in vitro. Pharm Res.

[74] Oude Elferink RP, Zadina J (2001). MDR1 P-glycoprotein transports endogenous opioid peptides. Peptides..

[75] Cserr H (1965). Potassium exchange between cerebrospinal fluid, plasma, and brain. Am J Phys.

[76] Bass NH, Lundborg P (1973). Postnatal development of bulk flow in the cerebrospinal fluid system of the albino rat: clearance of carboxyl-( 14 C)inulin after intrathecal infusion. Brain Res.

[77] Sugiyama D, Kusuhara H, Shitara Y, Abe T, Meier PJ, Sekine T, Endou H, Suzuki H, Sugiyama Y (2001). Characterization of the efflux transport of 17beta-estradiol-D-17beta-glucuronide from the brain across the blood-brain barrier. J Pharmacol Exp Ther.

[78] Noé B, Hagenbuch B, Stieger B, Meier PJ (1997). Isolation of a multispecific organic anion and cardiac glycoside transporter from rat brain. Proc Natl Acad Sci U S A.

[79] Uchino H, Kanai Y, Kim DK, Wempe MF, Chairoungdua A, Morimoto E, Anders MW, Endou H (2002). Transport of amino acid-related compounds mediated by L-type amino acid transporter 1 (LAT1): insights into the mechanisms of substrate recognition. Mol Pharmacol.

[80] Kalliokoski A, Niemi M (2009). Impact of OATP transporters on pharmacokinetics. Br J Pharmacol.

[81] Roth M, Obaidat A, Hagenbuch B (2012). OATPs, OATs and OCTs: the organic anion and cation transporters of the SLCO and SLC22A gene superfamilies. Br J Pharmacol.

[82] Wishart DS, Knox C, Guo AC, Shrivastava S, Hassanali M, Stothard P, Chang Z, Woolsey J (2006). DrugBank: a comprehensive resource for in silico drug discovery and exploration. Nucleic Acids Res.

[83] Kesselheim AS, Hwang TJ, Franklin JM (2015). Two decades of new drug development for central nervous system disorders. Nat Rev Drug Discov.

[84] Pankevich DE, Altevogt BM, Dunlop J, Gage FH, Hyman SE (2014). Improving and accelerating drug development for nervous system disorders. Neuron..

[85] Pardridge WM, Oldendorf WH (1975). Kinetic analysis of blood-brain barrier transport of amino acids. Biochim Biophys Acta Biomembr.

[86] Yahr MD, Duvoisin RC, Schear MJ, Barrett RE, Hoehn MM (1969). Treatment of parkinsonism with levodopa. Arch Neurol.

[87] Marsden CD, Barry PE, Parkes JD, Zilkha KJ (1973). Treatment of Parkinson's disease with levodopa combined with L-alpha-methyldopahydrazine, an inhibitor of extracerebral DOPA decarboxylase. J Neurol Neurosurg Psychiatry.

[88] Keränen T, Gordin A, Harjola VP, Karlsson M, Korpela K, Pentikäinen PJ, Rita H, Seppälä L, Wikberg T (1993). The effect of catechol-O-methyl transferase inhibition by entacapone on the pharmacokinetics and metabolism of levodopa in healthy volunteers. Clin Neuropharmacol.

[89] Huttunen KM, Raunio H, Rautio J (2011). Prodrugs--from serendipity to rational design. Pharmacol Rev.

[90] Rautio J, Meanwell NA, Di L, Hageman MJ (2018). The expanding role of prodrugs in contemporary drug design and development. Nat Rev Drug Discov.

[91] Olesen J, Gustavsson A, Svensson M, Wittchen HU, Jonsson B (2012). The economic cost of brain disorders in Europe. Eur J Neurol.

[92] Gynther M, Peura L, Vernerova M, Leppanen J, Karkkainen J, Lehtonen M, Rautio J, Huttunen KM (2016). Amino acid Promoieties Alter Valproic acid pharmacokinetics and enable extended brain exposure. Neurochem Res.

[93] Gynther M, Pickering DS, Spicer JA, Denny WA, Huttunen KM (2016). Systemic and brain pharmacokinetics of perforin inhibitor prodrugs. Mol Pharm.

[94] Puris E, Gynther M, Huttunen J, Petsalo A, Huttunen KM (2017). L-type amino acid transporter 1 utilizing prodrugs: how to achieve effective brain delivery and low systemic exposure of drugs. J Control Release.

[95] Montaser AB, Järvinen J, Löffler S, Huttunen J, Auriola S, Lehtonen M, Jalkanen A, Huttunen KM (2020). L-type amino acid transporter 1 enables the efficient brain delivery of small-sized prodrug across the blood-brain barrier and into human and mouse brain parenchymal cells. ACS Chem Neurosci.

[96] Puris E, Gynther M, Huttunen J, Auriola S, Huttunen KM (2019). L-type amino acid transporter 1 utilizing prodrugs of ferulic acid revealed structural features supporting the design of prodrugs for brain delivery. Eur J Pharm Sci.

[97] Huttunen J, Peltokangas S, Gynther M, Natunen T, Hiltunen M, Auriola S, Ruponen M, Vellonen KS, Huttunen KM (2019). L-type amino acid transporter 1 (LAT1/Lat1)-utilizing prodrugs can improve the delivery of drugs into neurons, astrocytes and microglia. Sci Rep.

[98] Puris E, Gynther M, de Lange ECM, Auriola S, Hammarlund-Udenaes M, Huttunen KM, Loryan I (2019). Mechanistic study on the use of the l-type amino acid transporter 1 for brain intracellular delivery of Ketoprofen via prodrug: A novel approach supporting the development of prodrugs for intracellular targets. Mol Pharm.

[99] Gynther M, Puris E, Peltokangas S, Auriola S, Kanninen KM, Koistinaho J, Huttunen KM, Ruponen M, Vellonen KS (2018). Alzheimer's disease phenotype or inflammatory insult does not Alter function of L-type amino acid transporter 1 in mouse blood-brain barrier and primary astrocytes. Pharm Res.

[100] Montaser A, Huttunen J, Ibrahim SA, Huttunen KM (2019). Astrocyte-targeted transporter-utilizing derivatives of Ferulic acid can have multifunctional effects ameliorating inflammation and oxidative stress in the brain. Oxidative Med Cell Longev.

[101] Montaser A, Lehtonen M, Gynther M, Huttunen KM. L-type amino acid transporter 1-utilizing prodrugs of Ketoprofen can efficiently reduce brain prostaglandin levels. Pharmaceutics. 2020;12(4). 10.3390/pharmaceutics12040344 PMC7238114 32290494

[102] Tampio J, Huttunen J, Montaser A, Huttunen KM. Targeting of perforin inhibitor into the brain parenchyma via a prodrug approach can decrease oxidative stress and Neuroinflammation and improve cell survival. Mol Neurobiol. 2020. 10.1007/s12035-020-02045-7 PMC7515946 32754897

[103] Tampio J, Löffler S, Guillon M, Hugele A, Huttunen J, Huttunen KM (2021). Improved l-type amino acid transporter 1 (LAT1)-mediated delivery of anti-inflammatory drugs into astrocytes and microglia with reduced prostaglandin production. Int J Pharm.

[104] Huttunen KM (2018). Identification of human, rat and mouse hydrolyzing enzymes bioconverting amino acid ester prodrug of ketoprofen. Bioorg Chem.

[105] Jalkanen AJ, Ihalainen J, Lehtonen M, Forsberg MM, Rautio J, Huttunen KM, Gynther M (2021). Species differences in the intra-brain distribution of an L-type amino acid transporter 1 (LAT1) -utilizing compound between mice and rats. Int J Pharm.

[106] Prasad PD, Wang H, Huang W, Kekuda R, Rajan DP, Leibach FH, Ganapathy V (1999). Human LAT1, a subunit of system L amino acid transporter: molecular cloning and transport function. Biochem Biophys Res Commun.

[107] Kanai Y, Segawa H, Miyamoto K, Uchino H, Takeda E, Endou H (1998). Expression cloning and characterization of a transporter for large neutral amino acids activated by the heavy chain of 4F2 antigen (CD98). J Biol Chem.

[108] Boado RJ, Li JY, Nagaya M, Zhang C, Pardridge WM (1999). Selective expression of the large neutral amino acid transporter at the blood-brain barrier. Proc Natl Acad Sci U S A.

[109] Zhao Y, Qu B, Wu X, Li X, Liu Q, Jin X, Guo L, Hai L, Wu Y (2014). Design, synthesis and biological evaluation of brain targeting l-ascorbic acid prodrugs of ibuprofen with “lock-in” function. Eur J Med Chem.

[110] Subudhi BB, Sahu PK, Singh VK, Prusty S (2018). Conjugation to ascorbic acid enhances brain availability of losartan carboxylic acid and protects against parkinsonism in rats. AAPS J.

[111] Schlessinger A, Welch MA, van Vlijmen H, Korzekwa K, Swaan PW, Matsson P (2018). Molecular modeling of drug-transporter interactions-an international transporter consortium perspective. Clin Pharmacol Ther.

[112] Seeger MA (2018). Membrane transporter research in times of countless structures. Biochim Biophys Acta Biomembr.

[113] Januliene D, Moeller A (2020). Cryo-EM of ABC transporters: an ice-cold solution to everything?. FEBS Lett.

[114] Majumder P, Mallela AK, Penmatsa A (2018). Transporters through the looking glass. An insight into the mechanisms of ion-coupled transport and methods that help reveal them. J Indian Inst Sci.

[115] Colas C, Ung PM, Schlessinger A (2016). SLC transporters: structure, function, and drug discovery. Medchemcomm..

[116] Senior AW, Evans R, Jumper J, Kirkpatrick J, Sifre L, Green T, Qin C, Žídek A, Nelson AWR, Bridgland A, Penedones H, Petersen S, Simonyan K, Crossan S, Kohli P, Jones DT, Silver D, Kavukcuoglu K, Hassabis D (2020). Improved protein structure prediction using potentials from deep learning. Nature..

[117] Heo L, Feig M (2020). High-accuracy protein structures by combining machine-learning with physics-based refinement. Proteins..

[118] Schlick T, Portillo-Ledesma S (2021). Biomolecular modeling thrives in the age of technology. Nat Comput Sci.

[119] Noé F, Tkatchenko A, Müller KR, Clementi C (2020). Machine learning for molecular simulation. Annu Rev Phys Chem.

[120] Drew D, North RA, Nagarathinam K, Tanabe M (2021). Structures and general transport mechanisms by the major facilitator superfamily (MFS). Chem Rev.

[121] Forrest LR, Rudnick G (2009). The rocking bundle: a mechanism for ion-coupled solute flux by symmetrical transporters. Physiology (Bethesda).

[122] Garaeva AA, Slotboom DJ (2020). Elevator-type mechanisms of membrane transport. Biochem Soc Trans.

[123] Lee Y, Wiriyasermkul P, Jin C, Quan L, Ohgaki R, Okuda S, Kusakizako T, Nishizawa T, Oda K, Ishitani R, Yokoyama T, Nakane T, Shirouzu M, Endou H, Nagamori S, Kanai Y, Nureki O (2019). Cryo-EM structure of the human L-type amino acid transporter 1 in complex with glycoprotein CD98hc. Nat Struct Mol Biol.

[124] Yan R, Zhao X, Lei J, Zhou Q (2019). Structure of the human LAT1-4F2hc heteromeric amino acid transporter complex. Nature..

[125] Ylikangas H, Peura L, Malmioja K, Leppänen J, Laine K, Poso A, Lahtela-Kakkonen M, Rautio J (2013). Structure-activity relationship study of compounds binding to large amino acid transporter 1 (LAT1) based on pharmacophore modeling and in situ rat brain perfusion. Eur J Pharm Sci.

[126] Ylikangas H, Malmioja K, Peura L, Gynther M, Nwachukwu EO, Leppanen J, Laine K, Rautio J, Lahtela-Kakkonen M, Huttunen KM, Poso A. Quantitative insight into the Design of Compounds Recognized by the L-type amino acid transporter 1 (LAT1). ChemMedChem. 2014. 10.1002/cmdc.201402281 25205473

[127] Huttunen J, Gynther M, Vellonen KS, Huttunen KM (2019). L-type amino acid transporter 1 (LAT1)-utilizing prodrugs are carrier-selective despite having low affinity for organic anion transporting polypeptides (OATPs). Int J Pharm.

[128] Karkkainen J, Gynther M, Kokkola T, Petsalo A, Auriola S, Lahtela-Kakkonen M, Laine K, Rautio J, Huttunen KM (2018). Structural properties for selective and efficient l-type amino acid transporter 1 (LAT1) mediated cellular uptake. Int J Pharm.

[129] Huttunen KM, Huttunen J, Aufderhaar I, Gynther M, Denny WA, Spicer JA (2016). L-type amino acid transporter 1 (lat1)-mediated targeted delivery of perforin inhibitors. Int J Pharm.

[130] Markowicz-Piasecka M, Huttunen J, Montaser A, Huttunen KM (2020). Hemocompatible LAT1-inhibitor can induce apoptosis in cancer cells without affecting brain amino acid homeostasis. Apoptosis..

[131] Milkereit R, Persaud A, Vanoaica L, Guetg A, Verrey F, Rotin D (2015). LAPTM4b recruits the LAT1-4F2hc Leu transporter to lysosomes and promotes mTORC1 activation. Nat Commun.

[132] Lai Y, Tse CM, Unadkat JD (2004). Mitochondrial expression of the human equilibrative nucleoside transporter 1 (hENT1) results in enhanced mitochondrial toxicity of antiviral drugs. J Biol Chem.

[133] Lee EW, Lai Y, Zhang H, Unadkat JD (2006). Identification of the mitochondrial targeting signal of the human equilibrative nucleoside transporter 1 (hENT1): implications for interspecies differences in mitochondrial toxicity of fialuridine. J Biol Chem.

[134] Hu C, Tao L, Cao X, Chen L (2020). The solute carrier transporters and the brain: physiological and pharmacological implications. Asian J Pharm Sci.

[135] Lin L, Yee SW, Kim RB, Giacomini KM (2015). SLC transporters as therapeutic targets: emerging opportunities. Nat Rev Drug Discov.

[136] Matović J, Järvinen J, Sokka IK, Imlimthan S, Raitanen JE, Montaser A, Maaheimo H, Huttunen KM, Peräniemi S, Airaksinen AJ, Sarparanta M, Johansson MP, Rautio J, Ekholm FS (2021). Exploring the biochemical foundations of a successful GLUT1-targeting strategy to BNCT: chemical synthesis and in vitro evaluation of the entire positional isomer library of ortho-Carboranylmethyl-bearing Glucoconjugates. Mol Pharm.

[137] Zhang K, Xu P, Sowers JL, Machuca DF, Mirfattah B, Herring J, Tang H, Chen Y, Tian B, Brasier AR, Sowers LC (2017). Proteome analysis of hypoxic glioblastoma cells reveals sequential metabolic adaptation of one-carbon metabolic pathways. Mol Cell Proteomics.

[138] Ohnishi K, Misawa M, Sikano N, Nakai K, Suzuki M (2021). Enhancement of Cancer cell-killing effects of boron neutron capture therapy by manipulating the expression of L-type amino acid transporter 1. Radiat Res.

[139] Uchida Y, Higuchi T, Shirota M, Kagami S, Saigusa D, Koshiba S, Yasuda J, Tamiya G, Kuriyama S, Kinoshita K, Yaegashi N, Yamamoto M, Terasaki T, Sugawara J (2021). Identification and validation of combination plasma biomarker of Afamin, fibronectin and sex hormone-binding globulin to predict pre-eclampsia. Biol Pharm Bull.

[140] Mochizuki T, Mizuno T, Maeda K, Kusuhara H (2021). Current progress in identifying endogenous biomarker candidates for drug transporter phenotyping and their potential application to drug development. Drug Metab Pharmacokinet.

[141] Müller F, Sharma A, König J, Fromm MF (2018). Biomarkers for in vivo assessment of transporter function. Pharmacol Rev.

[142] Stopfer P, Giessmann T, Hohl K, Sharma A, Ishiguro N, Taub ME, Zimdahl-Gelling H, Gansser D, Wein M, Ebner T, Müller F (2016). Pharmacokinetic evaluation of a drug transporter cocktail consisting of digoxin, furosemide, metformin, and Rosuvastatin. Clin Pharmacol Ther.

[143] Prueksaritanont T, Chu X, Evers R, Klopfer SO, Caro L, Kothare PA, Dempsey C, Rasmussen S, Houle R, Chan G, Cai X, Valesky R, Fraser IP, Stoch SA (2014). Pitavastatin is a more sensitive and selective organic anion-transporting polypeptide 1B clinical probe than rosuvastatin. Br J Clin Pharmacol.

[144] Ogasawara K, Wood-Horrall RN, Thomas M, Liu L, Liu M, Xue Y, Surapaneni S, Carayannopoulos LN, Zhou S, Palmisano M, Krishna G (2021). Impact of fedratinib on the pharmacokinetics of transporter probe substrates using a cocktail approach. Cancer Chemother Pharmacol.

[145] Li Y, Talebi Z, Chen X, Sparreboom A, Hu S. Endogenous biomarkers for SLC transporter-mediated drug-drug interaction evaluation. Molecules. 2021;26(18). 10.3390/molecules26185500 PMC8466752 34576971

[146] Xiao Y, Deng J, Liu X, Huang J, Sun Y, Dai R, Hong M (2014). Different binding sites of bovine organic anion-transporting polypeptide1a2 are involved in the transport of different fluoroquinolones. Drug Metab Dispos.

[147] Maeda T, Takahashi K, Ohtsu N, Oguma T, Ohnishi T, Atsumi R, Tamai I (2007). Identification of influx transporter for the quinolone antibacterial agent levofloxacin. Mol Pharm.

[148] Bednarczyk D, Sanghvi MV (2020). Organic anion transporting polypeptide 2B1 (OATP2B1), an expanded substrate profile, does it align with OATP2B1's hypothesized function?. Xenobiotica..

[149] Bailey DG, Dresser GK, Leake BF, Kim RB (2007). Naringin is a major and selective clinical inhibitor of organic anion-transporting polypeptide 1A2 (OATP1A2) in grapefruit juice. Clin Pharmacol Ther.

[150] Shirasaka Y, Suzuki K, Nakanishi T, Tamai I (2011). Differential effect of grapefruit juice on intestinal absorption of statins due to inhibition of organic anion transporting polypeptide and/or P-glycoprotein. J Pharm Sci.

[151] Brzica H, Abdullahi W, Reilly BG, Ronaldson PT (2018). Sex-specific differences in organic anion transporting polypeptide 1a4 (Oatp1a4) functional expression at the blood-brain barrier in Sprague-Dawley rats. Fluids Barriers CNS.

[152] Taniguchi T, Zanetti-Yabur A, Wang P, Usyk M, Burk RD, Wolkoff AW (2020). Interindividual diversity in expression of organic anion uptake transporters in Normal and cirrhotic human liver. Hepatol Commun.

[153] Yao J, Hong W, Huang J, Zhan K, Huang H, Hong M (2012). N-glycosylation dictates proper processing of organic anion transporting polypeptide 1B1. PLoS One.

[154] Clarke JD, Novak P, Lake AD, Hardwick RN, Cherrington NJ (2017). Impaired N-linked glycosylation of uptake and efflux transporters in human non-alcoholic fatty liver disease. Liver Int.

[155] Hong M, Hong W, Ni C, Huang J, Zhou C (2015). Protein kinase C affects the internalization and recycling of organic anion transporting polypeptide 1B1. Biochim Biophys Acta.

[156] Mayati A, Le Vee M, Moreau A, Jouan E, Bucher S, Stieger B, Denizot C, Parmentier Y, Fardel O (2015). Protein kinase C-dependent regulation of human hepatic drug transporter expression. Biochem Pharmacol.

[157] Bludau I, Aebersold R (2020). Proteomic and interactomic insights into the molecular basis of cell functional diversity. Nat Rev Mol Cell Biol.

[158] Logan RW, McClung CA (2019). Rhythms of life: circadian disruption and brain disorders across the lifespan. Nat Rev Neurosci.

[159] Esposito E, Li W, E TM, Park JH, Sencan I, Guo S, Shi J, Lan J, Lee J, Hayakawa K, Sakadzic S, Ji X, Lo EH. (2020). Potential circadian effects on translational failure for neuroprotection. Nature..

[160] Pulido RS, Munji RN, Chan TC, Quirk CR, Weiner GA, Weger BD, Rossi MJ, Elmsaouri S, Malfavon M, Deng A, Profaci CP, Blanchette M, Qian T, Foreman KL, Shusta EV, Gorman MR, Gachon F, Leutgeb S, Daneman R (2020). Neuronal activity regulates blood-brain barrier efflux transport through endothelial circadian genes. Neuron..

[161] Zhang SL, Lahens NF, Yue Z, Arnold DM, Pakstis PP, Schwarz JE, Sehgal A (2021). A circadian clock regulates efflux by the blood-brain barrier in mice and human cells. Nat Commun.

[162] Kervezee L, Hartman R, van den Berg DJ, Shimizu S, Emoto-Yamamoto Y, Meijer JH, de Lange EC (2014). Diurnal variation in P-glycoprotein-mediated transport and cerebrospinal fluid turnover in the brain. AAPS J.

[163] Munro LJ, Kell DB (2021). Intelligent host engineering for metabolic flux optimisation in biotechnology. Biochem J.

