# Supplementary material for: Pharmacoproteomics of Brain Barrier Transporters and Substrate Design for the Brain Targeted Drug Delivery
Source: Pharm Res. 2022 Mar 7;39(7):1363–92. doi: 10.1007/s11095-022-03193-2 (PMC9246989; doi:10.1007/s11095-022-03193-2)
Supplement: Supplementary file 1 — (PDF 491 kb) [file 11095_2022_3193_MOESM1_ESM.pdf]

Supporting Information

Pharmacoproteomics of Brain Barrier Transporters and Substrate Design for the Brain Targeted Drug Delivery

Kristiina M. Huttunen[1,], Tetsuya Terasaki[1,**], Arto Urtti[1], Ahmed B. Montaser[1], Yasuo Uchida[2]*

[1] School of Pharmacy, Faculty of Health Sciences, University of Eastern Finland,

P.O. Box 1627, FI-70211 Kuopio, Finland

[2]Graduate School of Pharmaceutical Sciences, Tohoku University,

6-3 Aoba, Aramaki, Aoba-ku, Sendai, 980-8578, Japan

* Corresponding author, E-mail: kristiina.huttunen@uef.fi, ORCID: 0000-0002-1175-8517

** Corresponding author, E-mail: tetsuya.terasaki@uef.fi, ORCID: 0000-0002-6332-7575

Contents

Table S1. Prediction of $K_{p,uu,brain,MDR1,human}$ of MDR1 substrate in human. 2
Table S2. Prediction of $K_{p,uu,brain,BCRP,human}$ of BCRP substrate in human.................................3

Table S1. Prediction of $K_{p,uu,brain,MDR1,human}$ of MDR1 substrate in human.

Rat<br>$K_{p,uu,brain,MDR1,rat}$	Rat<br>$TA_{vivo,MDR1,rat}P_{passive,vivo,MDR1,rat}$	Human<br>$K_{p,uu,brain,MDR1,human}$	Ratio<br>$K_{p,uu,brain,MDR1,human}/K_{p,uu,brain,MDR1,rat}$
1.00×10^{-4}	20.1	9.87×10^{-4}	9.87
5.00×10^{-4}	4.02	4.92×10^{-3}	9.84
1.00×10^{-3}	2.01	9.79×10^{-3}	9.79
5.00×10^{-3}	4.00×10^{-1}	4.73×10^{-2}	9.46
1.00×10^{-2}	1.99×10^{-1}	9.07×10^{-2}	9.07
2.00×10^{-2}	9.86×10^{-2}	1.68×10^{-1}	8.39
3.00×10^{-2}	6.51×10^{-2}	2.34×10^{-1}	7.80
4.00×10^{-2}	4.83×10^{-2}	2.92×10^{-1}	7.29
5.00×10^{-2}	3.82×10^{-2}	3.42×10^{-1}	6.84
6.00×10^{-2}	3.15×10^{-2}	3.86×10^{-1}	6.45
7.00×10^{-2}	2.67×10^{-2}	4.27×10^{-1}	6.09
8.00×10^{-2}	2.31×10^{-2}	4.62×10^{-1}	5.78
9.00×10^{-2}	2.03×10^{-2}	4.94×10^{-1}	5.49
1.00×10^{-1}	1.81×10^{-2}	5.23×10^{-1}	5.23
2.00×10^{-1}	8.05×10^{-3}	7.12×10^{-1}	3.56
3.00×10^{-1}	4.69×10^{-3}	8.09×10^{-1}	2.70
4.00×10^{-1}	3.02×10^{-3}	8.68×10^{-1}	2.17
5.00×10^{-1}	2.01×10^{-3}	9.08×10^{-1}	1.81
6.00×10^{-1}	1.34×10^{-3}	9.37×10^{-1}	1.56
7.00×10^{-1}	8.62×10^{-4}	9.58×10^{-1}	1.37
8.00×10^{-1}	5.03×10^{-4}	9.75×10^{-1}	1.22
9.00×10^{-1}	2.24×10^{-4}	9.89×10^{-1}	1.10
9.90×10^{-1}	2.03×10^{-5}	9.99×10^{-1}	1.01

Changing $K_{p,uu,brain,MDR1,rat}$ from 1.00×10^{-4} to 9.90×10^{-1} with $PEL_{MDR1,rat}$ of rat cerebrum (497 fmol/cm^2), $TA_{MDR1, rat, vivo}/P_{passive,rat,vivo}$ was estimated by Eq. 5. Assuming $RTA_{MDR1,human/rat}$ is 1.0, $K_{p,uu,brain,MDR1,human}$ was predicted by Eq. 7 with $PEL_{MDR1,human}$ of human cerebral cortex (50.3 fmol/cm^2).

Table S2. Prediction of $K_{p,uu,brain,BCRP,human}$ of BCRP substrate in human.

Rat $K_{p,uu,brain,BCRP,rat}$	Rat $TA_{vivo,BCRP,rat}/P_{passive,vivo,BCRP,rat}$	Human $K_{p,uu,brain,BCRP,human}$	Ratio $K_{p,uu,brain,BCRP,,human}/K_{p,uu,brain,BCRP,rat}$
1.00 x 10^{-4}	46.1	5.23 x 10^{-4}	5.23
5.00 x 10^{-4}	9.21	2.61 x 10^{-3}	5.22
1.00 x 10^{-3}	4.60	5.21 x 10^{-3}	5.21
5.00 x 10^{-3}	9.17 x 10^{-1}	2.56 x 10^{-2}	5.12
1.00 x 10^{-2}	4.56 x 10^{-1}	5.02 x 10^{-2}	5.02
2.00 x 10^{-2}	2.26 x 10^{-1}	9.64 x 10^{-2}	4.82
3.00 x 10^{-2}	1.49 x 10^{-1}	1.39 x 10^{-1}	4.64
4.00 x 10^{-2}	1.11 x 10^{-1}	1.79 x 10^{-1}	4.47
5.00 x 10^{-2}	8.76 x 10^{-2}	2.16 x 10^{-1}	4.32
6.00 x 10^{-2}	7.22 x 10^{-2}	2.50 x 10^{-1}	4.17
7.00 x 10^{-2}	6.12 x 10^{-2}	2.82 x 10^{-1}	4.03
8.00 x 10^{-2}	5.30 x 10^{-2}	3.13 x 10^{-1}	3.91
9.00 x 10^{-2}	4.66 x 10^{-2}	3.41 x 10^{-1}	3.79
1.00 x 10^{-1}	4.15 x 10^{-2}	3.67 x 10^{-1}	3.67
2.00 x 10^{-1}	1.84 x 10^{-2}	5.67 x 10^{-1}	2.83
3.00 x 10^{-1}	1.08 x 10^{-2}	6.91 x 10^{-1}	2.31
4.00 x 10^{-1}	6.91 x 10^{-3}	7.77 x 10^{-1}	1.94
5.00 x 10^{-1}	4.61 x 10^{-3}	8.39 x 10^{-1}	1.68
6.00 x 10^{-1}	3.07 x 10^{-3}	8.87 x 10^{-1}	1.48
7.00 x 10^{-1}	1.97 x 10^{-3}	9.24 x 10^{-1}	1.32
8.00 x 10^{-1}	1.15 x 10^{-3}	9.54 x 10^{-1}	1.19
9.00 x 10^{-1}	5.12 x 10^{-4}	9.79 x 10^{-1}	1.09
9.90 x 10^{-1}	4.65 x 10^{-5}	9.98 x 10^{-1}	1.01

Changing $K_{p,uu,brain,BCRP,rat}$ from 1.00 x 10^{-4} to 9.90 x 10^{-1} with $PEL_{BCRP,rat}$ of rat cerebrum (217 fmol/cm^2), $TA_{BCRP, rat, vivo}/P_{passive,rat,vivo}$ was estimated by Eq. 5. Assuming $RTA_{BCRP, human/rat}$ is 1.0, $K_{p,uu,brain,BCRP,human}$ was predicted by Eq. 7 with $PEL_{BCRP,human}$ of human cerebral cortex (41.5 fmol/cm^2).
